# Supplementary material for: Simultaneous multiple allelic replacement in the malaria parasite enables dissection of PKG function
Source: Life Sci Alliance. 2020 Mar 16;3(4):e201900626. doi: 10.26508/lsa.201900626 (PMC7081069; doi:10.26508/lsa.201900626)
Supplement: Supplementary file 3 [file LSA-2019-00626_Supplemental_Data_1.pdf]

Simultaneous multiple allelic replacement in the malaria parasite enables dissection of PKG function

Dataset

Dataset 1 – Integration constructs used in this study Page 2

pMX_2lox (1023 bp)

GAATGAAAGAAATAAAAAGAAGGCTATATTTTCAAATGATGATTTTACAGGAGAAGATAGTTTAATGGAGgtaagaaataaaaggaatttgtaatttttttttaaattgtatatttaaaaggga
CTTACTTTCTTTATTTTTCTTCCGATATAAAAGTTTACTACTAAAATGTCCTCTTCTATCAAATTACCTCcattctttattttccttaaacattaaaaaaaaatttaacatataaattttccct

5’ homolgy arm

20 40 60 80 100 120

ataatgtatgtattaattttttgtgatcgtttattatatattatttttaaattacatatccatatttaattatatacaaatgttgttttcagaatttttttttttttttttttctatttatctata
tattacatacataattaaaaacactagcaaataatatataataaaaatttaatgtataggtataaattaatatatgtttacaacaaaagtcttaaaaaaaaaaaaaaaaaagataaatagatat

5’ homolgy arm

140 160 180 200 220 240

acatatatttaattttattttttagGATCATTTAGAACTTCGGGAAAAGCTTTCAGAAGATATTGATATGATAAAGACTTCCTTAAAAAATAATCTAGTTTGTAGTACATTAAACGATAATGAAAT
tgtatataaattaaaataaaaatcCTAGTAAATCTTGAAGCCCTTTTCGAAAGTCTTCTATAACTATACTATTTCTGAAGGAATTTTTTATTAGATCAAACATCATGTAATTTGCTATTACTTTA

5’ homolgy arm

260 280 300 320 340 360

ATTGACTCTGTCTAATTATATGCAATTCTTTGTTTTTAAAAGTGGAAATTTAGTAATAAAACAAGGGGAAAAAGgtaaataaaaaaaataatatacaATAACTTCGTATAGTATACCTTATACGA
TAACTGAGACAGATTAATATACGTTAAGAAACAAAAATTTTCACCTTTAAATCATTATTTTGTTCCCCTTTTTCcatttattttttttattatatgtTATTGAAGCATATCATATGGAATATGCT

Lox N site

5’ homolgy arm

380 400 420 440 460 480 500

AGTTATtatatatgtatatatataATAACTTCGTATAGGATACTTTATACGAAGTTATtatatttatatattttatattcttttagGGTCATACTTTTTCATTATTAATAGTGGCAAATTTGACG
TCAATAatatatacatatatatatTATTGAAGCATATCCTATGAAATATGCTTCAATAatataaatatataaaatataagaaaatcCCAGTATGAAAAAGTAATAATTATCACCGTTTAAACTGC

Lox 2272

2loxPint (sera2 intron with loxN and lox2272)

3’ homology arm

520 540 560 580 600 620

TTTATGTAAATGATAAAAAAGTAAAGACTATGGGAAAAGGTAGTTCTTTCGGTGAAGCTGCTTTAATTCATAATACCCAAAGAAGTGCAACTATTATTGCAGAAACTGATGGAACTCTATGGGGA
AAATACATTTACTATTTTTTCATTTCTGATACCCTTTTCCATCAAGAAAGCCACTTCGACGAAATTAAGTATTATGGGTTTCTTCACGTTGATAATAACGTCTTTGACTACCTTGAGATACCCCT

3’ homology arm

640 660 680 700 720 740

GTTCAAAGAAGTACATTTAGAGCTACCCTAAAACAATTATCTAATAGAAATTTTAACGAAAACAGAACATTTATCGATTCCGTTTCAGTTTTTGATATGTTAACTGAAGCACAAAAAAACATGAT
CAAGTTTCTTCATGTAAATCTCGATGGGATTTTGTTAATAGATTATCTTTAAAATTGCTTTTGTCTTGTAAATAGCTAAGGCAAAGTCAAAAACTATACAATTGACTTCGTGTTTTTTTGTACTA

3’ homology arm

760 780 800 820 840 860

TACTAATGCTTGTGTAATACAAAACTTTAAATCTGGTGAAACCATTGTTAAACAAGGAGATTATGGAGATGTCTTATACATTTTGAAAGAAGGAAAGGCTACAGTATATATTAACGATGAAGAGA
ATGATTACGAACACATTATGTTTTGAAATTTAGACCACTTTGGTAACAATTTGTTCCTCTAATACCTCTACAGAATATGTAAAACTTTCTTCCTTTCCGATGTCATATATAATTGCTACTTCTCT

3’ homology arm

880 900 920 940 960 980 1,000

TAAGGGTTTTAGAGAAAgtacgc
ATTCCCAAAATCTCTTTcatgcg

3’ homology arm

1,010 1,020

pDC_loxNPKG:lox22mCherry (7298 bp)

TATTTTCTAACAGAATTAGTAACAGGTGGAGAATTATATGATGCTATTAGAAAATTAGGTTTATTATCTAAATCACAAGCTCAATTTTATTTAGGTTCTATCATTTTAGCTATTGAATATTTACA
ATAAAAGATTGTCTTAATCATTGTCCACCTCTTAATATACTACGATAATCTTTTAATCCAAATAATAGATTTAGTGTTCGAGTTAAAATAAATCCAAGATAGTAAAATCGATAACTTATAAATGT

5' homology arm

20 40 60 80 100 120

TGAAAGAAATATTGTATATAGAGATTTAAAACCAGAAAACATTTTATTAGATAAACAAGGTTATGTAAAACTAATCGATTTTGGTTGTGCCAAAAAGGTACAAGGTAGAGCTTATACATTAGTAG
ACTTTCTTTATAACATATATCTCTAAATTTTGGTCTTTTGTAAAATAATCTATTTGTTCCAATACATTTTGATTAGCTAAAACCAACACGGTTTTTCCATGTTCCATCTCGAATATGTAATCATC

5' homology arm

140 160 180 200 220 240

GTACACCTCATTATATGGCACCTGAGGTTATTTTAGGAAAAGGTTATGGATGTACTGTTGACATATGGGCATTGGGAATATGCCTATATGAATTTATATGTGGTCCATTACCATTTGGTAATGAT
CATGTGGAGTAATATACCGTGGACTCCAATAAAATCCTTTTCCAATACCTACATGACAACTGTATACCCGTAACCCTTATACGGATATACTTAAATATACACCAGGTAATGGTAAACCATTACTA

5' homology arm

260 280 300 320 340 360

GAAGAAGATCAATTAGAAATTTTCagaGATATTCTGACAGGCCAGCTGACCTTTCCGGATTATGTTACCGATACCGATAGCATCAACCTGATGAAACGCCTGCTGTGTCGTCTGCCGCAGGGTCG
CTTCTTCTAGTTAATCTTTAAAAGtctCTATAAGACTGTCCGGTCGACTGGAAAGGCCTAATACAATGGCTATGGCTATCGTAGTTGGACTACTTTGCGGACGACACAGCAGACGGCGTCCCAGC

5' homology arm | pfpkgsynth

380 400 420 440 460 480 500

TATTGGTTGTAGCATTAATGGTTTTAAAGACATCAAAGATCACCCGTTCTTCAGCAACTTTAACTGGGATAAACTGGCAGGTCGTCTGCTGGACCCTCCGCTGGTTAGCAAAAGCGAAACCTATG
ATAACCAACATCGTAATTACCAAAATTTCTGTAGTTTCTAGTGGGCAAGAAGTCGTTGAAATTGACCCTATTTGACCGTCCAGCAGACGACCTGGGAGGCGACCAATCGTTTTCGCTTTGGATAC

pfpkgsynth

520 540 560 580 600 620

CAGAGGATATCGACATCAAACAAATCGAAGAAGAGGACGCAGAGGACGATGAGGAACCGCTGAATGACGAAGATAATTGGGATATCGATTTTTAAgaggttaccatcgagggatatggcagctta
GTCTCCTATAGCTGTAGTTTGTTTAGCTTCTTCTCCTGCGTCTCCTGCTACTCCTTGGCGACTTACTGCTTCTATTAACCCTATAGCTAAAAATTctccaatggtagctccctataccgtcgaat

pfpkgsynth | Pb DT 3'UTR

640 660 680 700 720 740

atgttcgtttttcttatttatatatttataccaattgattgtatttataactgtaaaaatgtgtatgttgtgtgcatatttttttttgtgcatgcacatgcatgtaaatagctaaaattatgaac
tacaagcaaaaagaataaatatataaatatggttaactaacataaatattgacatttttacacatacaacacacgtataaaaaaaaacacgtacgtgtacgtacatttatcgattttaatacttg

Pb DT 3'UTR

760 780 800 820 840 860

atttatttttgttcagaaaaaaaaaactttacacacataaaatggctagtatgaatagccatattttatataaattaaatcctatgaatttatgaccatattaaaaatttagatatttatgga
taaaataaaaacaagtcttttttttttgaaatgtgtgtattttaccgatcatacttatcggtataaaatatatttaatttaggatacttaaatactggtataatttttaaatctataaatacct

Pb DT 3'UTR

880 900 920 940 960 980 1,000

acataatatgtttgaaacaataagacaaaattattattattattattatttttactgttataattatgtgtctccttcaatgattcataaatagttggacttgattttaaaatgtttataatat
tgtattatacaaactttgttattctgttttaataataataataataataaaaatgacaatattaatacacagaggaagttactaagtatttatcaacctgaactaaaattttacaaatattata

Pb DT 3'UTR

1,020 1,040 1,060 1,080 1,100 1,120

```
gattagcatagttaaataaaaaaagttgaaaaattaaaaaaaaacatataaacacaaatgatggtttttccttcaatttcgatatcatatagcaatcgtattagcaggtaaatgagcaaggttaa
ctaatcgtatcaatttatttttttcaactttttaattttttttttgtatatttgtgtttactaccaaaaaggaagttaaagctatagtatatcgttagcataatcgtccatttactcgttccaatt
```
Pb DT 3'UTR

1,140 1,160 1,180 1,200 1,220 1,240

```
taaaaaaatggtaaaaaaaaaaaaattggggaaaaataaacaaggtgtctttatctacactttggctttacaatgtataatttatatgcatacaaaaaaattttgataaaattgtaaaaaggtgaa
atttttttaccatttttttttttttaaccccttttttatttgttccacagaaatagatgtgaaaccgaaatgttacatattaaatatacgtatgtttttttaaaactattttaacatttttccactt
```
Pb DT 3'UTR

1,260 1,280 1,300 1,320 1,340 1,360

```
taaaataacattgtaacagtagtagatagtaaagggaaggtgttgctcaaatagtgtcgaaacaaaactggcataaagaaaaattgatattgagcagaggatatgcgcataatggtattttgttt
attttattgtaacattgtcatcatctatcatttcccttccacaacgagtttatcacagctttgttttgaccgtatttctttttaactataactcgtctcctatacgcgtattaccataaaacaaa
```
Pb DT 3'UTR

1,380 1,400 1,420 1,440 1,460 1,480 1,500

```
gtttgtttgttttctcatttttttgagacagctcaattctttatgtccacaacatcatcggacttttcttcttcagggtaggcGGCCGCTCGAGCATGCATCTAGATTTTATATAATAAAAAATGG
caaacaaacaaaagagtaaaaaaactctgtcgagttaagaaatacaggtgttgtagtagcctgaaaagaagaagtcccatccgCCGGCGAGCTCGTACGTAGATCTAAAATATATTATTTTTTACC
```
Pb DT 3'UTR

1,520 1,540 1,560 1,580 1,600 1,620

```
AGAAGTAGAAATAGTAAAAAATAAAAAAAGGTTACgtgcggttaacaATAACTTCGTATAGTATACCTTATACGAAGTTATtatatatgtatatatatatatatttatatattttatattctttt
TCTTCATCTTTATCATTTTTTATTTTTTTCCAATGcacgccaattgtTATTGAAGCATATCATATGGAATATGCTTCAATAatatatacatatatatatatataaatatataaaatataagaaaa
```
loxN site SERA2 intron

1,640 1,660 1,680 1,700 1,720 1,740

```
agGCAGCTATTTCTTCATTATTAATAGCGGCAAATTTGATGTGTACGTGAACGACAAAAAAGTGAAAACCATGGGTAAAGGTAGCAGCTTTGGTGAAGCAGCACTGATTCATAATACCCAGCGTA
tcCGTCGATAAAGAAGTAATAATTATCGCCGTTTAAACTACACATGCACTTGCTGTTTTTTCACTTTTGGTACCCATTTCCATCGTCGAAACCACTTCGTCGTGACTAAGTATTATGGGTCGCAT
```
PKGsynth_cDNA

1,760 1,780 1,800 1,820 1,840 1,860

```
GCGCAACCATTATTGCAGAAACCGATGGCACCCTGTGGGGTGTTCAGCGTAGCACCTTTCGTGCAACCCTGAAACAGCTGAGCAATCGTAACTTTAATGAAAACCGCACCTTTATCGATAGCGTG
CGCGTTGGTAATAACGTCTTTGGCTACCGTGGGACACCCCACAAGTCGCATCGTGGAAAGCACGTTGGGACTTTGTCGACTCGTTAGCATTGAAATTACTTTTGGCGTGGAAATAGCTATCGCAC
```
PKGsynth_cDNA

1,880 1,900 1,920 1,940 1,960 1,980 2,000

```
AGCGTTTTTGATATGCTGACCGAAGCACAGAAAAACATGATTACCAATGCCTGCGTGATCCAGAACTTTAAAAGTGGTGAAACCATTGTGAAACAGGGCGATTATGGTGATGTTCTGTATATTCT
TCGCAAAAACTATACGACTGGCTTCGTGTCTTTTTGTACTAATGGTTACGGACGCACTAGGTCTTGAAATTTTCACCACTTTGGTAACACTTTGTCCCGCTAATACCACTACAAGACATATAAGA
```
PKGsynth_cDNA

2,020 2,040 2,060 2,080 2,100 2,120

```
GAAAGAGGGTAAAGCCACCGTCTATATCAATGATGAAGAGATTCGTGTGCTGGAAAAAGGCTCATATTTTGGTGAACGTGCACTGCTGTATGATGAACCGCGTTCAGCCACAATTATTGCAAAAG
CTTTCTCCCATTTCGGTGGCAGATATAGTTACTACTTCTCTAAGCACACGACCTTTTTCCGAGTATAAAACCACTTGCACGTGACGACATACTACTTGGCGCAAGTCGGTGTTAATAACGTTTTC
```
PKGsynth_cDNA

2,140 2,160 2,180 2,200 2,220 2,240

AACCGACCGCATGTGCAAGCATTTGTCGTAAACTGCTGAATATTGTTCTGGGCAATCTGCAGGTTGTTCTGTTTCGCAATATTATGACAGAAGCACTGCAGCAGAGCGAAATCTTTAAACAGTTT
TTGGCTGGCGTACACGTTCGTAAACAGCATTTGACGACTTATAACAAGACCCGTTAGACGTCCAACAAGACAAAGCGTTATAATACTGTCTTCGTGACGTCGTCTCGCTTTAGAAATTTGTCAAA

PKGsynth_cDNA

2,260 2,280 2,300 2,320 2,340 2,360

AGCGGTGATCAGCTGAATGATCTGGCAGATACCGCAATTGTTCGTGATTATCCGGCAAACTATAACATCCTGCACAAAGATAAAGTTAAAAGCGTGAAATACATCATCGTGCTGGAAGGTAAAGT
TCGCCACTAGTCGACTTACTAGACCGTCTATGGCGTTAACAAGCACTAATAGGCCGTTTGATATTGTAGGACGTGTTTCTATTTCAATTTTCGCACTTTATGTAGTAGCACGACCTTCCATTTCA

PKGsynth_cDNA

2,380 2,400 2,420 2,440 2,460 2,480 2,500

TGAACTGTTTCTGGATGATACCAGCATTGGTATTCTGAGCCGTGGTATGAGTTTTGGTGATCAGTATGTTCTGAATCAGAAACAGCCGTTCAAACACACCATTAAAAGCCTGGAAGTTTGCAAAA
ACTTGACAAAGACCTACTATGGTCGTAACCATAAGACTCGGCACCATACTCAAAACCACTAGTCATACAAGACTTAGTCTTTGTCGGCAAGTTTGTGTGGTAATTTTCGGACCTTCAAACGTTTT

PKGsynth_cDNA

2,520 2,540 2,560 2,580 2,600 2,620

TTGCCCTGATTACCGAAACCTGTCTGGCCGATTGTCTGGGTAATAACAATATTGATGCCAGCATCGATTACAATAACAAAAAATCCATCATCAAAAAAATGTACATCTTTCGCTATCTGACCGAT
AACGGGACTAATGGCTTTGGACAGACCGGCTAACAGACCCATTATTGTTATAACTACGGTCGTAGCTAATGTTATTGTTTTTTAGGTAGTAGTTTTTTTACATGTAGAAAGCGATAGACTGGCTA

PKGsynth_cDNA

2,640 2,660 2,680 2,700 2,720 2,740

AAACAGTGCAATCTGCTGATTGAAGCATTTCGTACCACCCGTTATGAAGAAGGCGATTATATCATTCAAGAAGGTGAAGTTGGCAGCCGCTTCTACATTATCAAAAATGGTGAAGTCGAAATCGT
TTTGTCACGTTAGACGACTAACTTCGTAAAGCATGGTGGGCAATACTTCTTCCGCTAATATAGTAAGTTCTTCCACTTCAACCGTCGGCGAAGATGTAATAGTTTTTACCACTTCAGCTTTAGCA

PKGsynth_cDNA

2,760 2,780 2,800 2,820 2,840 2,860

CAAAAACAAAAAACGTCTGCGTACCCTGGGCAAAAATGATTATTTTGGCGAGCGTGCCCTGCTGTACGACGAGCCTCGTACCGCAAGCGTTATTAGCAAAGTTAATAATGTGGAATGCTGGTTTG
GTTTTTGTTTTTTGCAGACGCATGGGACCCGTTTTTACTAATAAAACCGCTCGCACGGGACGACATGCTGCTCGGAGCATGGCGTTCGCAATAATCGTTTCAATTATTACACCTTACGACCAAAC

PKGsynth_cDNA

2,880 2,900 2,920 2,940 2,960 2,980 3,000

TGGACAAAAGCGTTTTTCTGCAGATTATTCAGGGTCCTATGCTGGCACATCTGGAAGAACGCATTAAAATGCAGGATACCAAAGTGGAAATGGATGAACTGGAAACCGAACGTATTATTGGTCGT
ACCTGTTTTCGCAAAAAGACGTCTAATAAGTCCCAGGATACGACCGTGTAGACCTTCTTGCGTAATTTTACGTCCTATGGTTTCACCTTTACCTACTTGACCTTTGGCTTGCATAATAACCAGCA

PKGsynth_cDNA

3,020 3,040 3,060 3,080 3,100 3,120

GGCACCTTTGGCACCGTTAAACTGGTGCATCACAAACCGACCAAAATTCGTTATGCACTGAAATGCGTTAGCAAACGCAGCATTATCAATCTGAACCAGCAGAACAACATTAAACTGGAACGTGA
CCGTGGAAACCGTGGCAATTTGACCACGTAGTGTTTGGCTGGTTTTAAGCAATACGTGACTTTACGCAATCGTTTGCGTCGTAATAGTTAGACTTGGTCGTCTTGTTGTAATTTGACCTTGCACT

PKGsynth_cDNA

3,140 3,160 3,180 3,200 3,220 3,240

AATTACCGCAGAAAACGATCATCCGTTTATTATCCGTCTGGTGCGTACCTTTAAAGATAGCAAATATTTCTACTTCCTGACCGAACTGGTTACCGGTGGTGAACTGTATGACGCAATTCGCAAAC
TTAATGGCGTCTTTTGCTAGTAGGCAAATAATAGGCAGACCACGCATGGAAATTTCTATCGTTTATAAAGATGAAGGACTGGCTTGACCAATGGCCACCACTTGACATACTGCGTTAAGCGTTTG

PKGsynth_cDNA

3,260 3,280 3,300 3,320 3,340 3,360

TGGGTCTGCTGAGCAAAAGCCAGGCACAGTTTTATCTGGGTAGCATTATTCTGGCCATCGAATATCTGCATGAACGCAATATTGTGTATCGTGATCTGAAACCGGAAAACATTCTGCTGGATAAA
ACCCAGACGACTCGTTTTCGGTCCGTGTCAAAATAGACCCATCGTAATAAGACCGGTAGCTTATAGACGTACTTGCGTTATAACACATAGCACTAGACTTTGGCCTTTTGTAAGACGACCTATTT

PKGsynth_cDNA

3,380 3,400 3,420 3,440 3,460 3,480 3,500

CAGGGCTATGTGAAACTGATTGATTTTGGGTGTGCCAAAAAAGTTCAGGGTCGTGCATATACCCTGGTTGGTACACCGCATTATATGGCACCGGAAGTTATTCTGGGAAAAGGTTATGGTTGTAC
GTCCCGATACACTTTGACTAACTAAAACCCACACGGTTTTTTCAAGTCCCAGCACGTATATGGGACCAACCATGTGGCGTAATATACCGTGGCCTTCAATAAGACCCTTTTCCAATACCAACATG

PKGsynth_cDNA

3,520 3,540 3,560 3,580 3,600 3,620

CGTTGATATTTGGGCACTGGGTATTTGTCTGTACGAATTTATCTGTGGTCCGCTGCCGTTTGGTAACGATGAAGAGGATCAGCTGGAAATTTTTCGTGATATTCTGACAGGCCAGCTGACCTTTC
GCAACTATAAACCCGTGACCCATAAACAGACATGCTTAAATAGACACCAGGCGACGGCAAACCATTGCTACTTCTCCTAGTCGACCTTTAAAAAGCACTATAAGACTGTCCGGTCGACTGGAAAG

PKGsynth_cDNA

3,640 3,660 3,680 3,700 3,720 3,740

CGGATTATGTTACCGATACCGATAGCATCAACCTGATGAAACGCCTGCTGTGTCGTCTGCCGCAGGGTCGTATTGGTTGTAGCATTAATGGTTTTAAAGACATCAAAGATCACCCGTTCTTCAGC
GCCTAATACAATGGCTATGGCTATCGTAGTTGGACTACTTTGCGGACGACACAGCAGACGGCGTCCCAGCATAACCAACATCGTAATTACCAAAATTTCTGTAGTTTCTAGTGGGCAAGAAGTCG

PKGsynth_cDNA

3,760 3,780 3,800 3,820 3,840 3,860

AACTTTAACTGGGATAAACTGGCAGGTCGTCTGCTGGACCCTCCGCTGGTTAGCAAAAGCGAAACCTATGCAGAGGATATCGACATCAAACAAATCGAAGAAGAGGACGCAGAGGACGATGAGGA
TTGAAATTGACCCTATTTGACCGTCCAGCAGACGACCTGGGAGGCGACCAATCGTTTTCGCTTTGGATACGTCTCCTATAGCTGTAGTTTGTTTAGCTTCTTCTCCTGCGTCTCCTGCTACTCCT

PKGsynth_cDNA

3,880 3,900 3,920 3,940 3,960 3,980 4,000

ACCGCTGAATGACGAAGATAATTGGGATATCGATTTTccTAgGTCTGTGAGTAAAGGAGAAGAACTTTTCACTGGAGTTGTCCCAATTCTTGTTGAATTAGATGGTGATGTTAATGGGCACAAAT
TGGCGACTTACTGCTTCTATTAACCCTATAGCTAAAAggATcCAGACACTCATTTCCTCTTCTTGAAAAGTGACCTCAACAGGGTTAAGAACAACTTAATCTACCACTACAATTACCCGTGTTTA

PKGsynth_cDNA eGFP

4,020 4,040 4,060 4,080 4,100 4,120

TTTCTGTCAGTGGAGAGGGTGAAGGTGATGCAACATACGGAAAACTTACCCTTAAATTTATTTGCACTACTGGAAAACTACCTGTTCCCTGGCCAACACTTGTCACTACTTTCGGTTATGGTGTT
AAAGACAGTCACCTCTCCCACTTCCACTACGTTGTATGCCTTTTGAATGGGAATTTAAATAAACGTGATGACCTTTTGATGGACAAGGGACCGGTTGTGAACAGTGATGAAAGCCAATACCACAA

eGFP

4,140 4,160 4,180 4,200 4,220 4,240

CAATGCTTTGCGAGATACCCAGATCACATGAAACAGCATGACTTTTTCAAGAGTGCCATGCCCGAAGGTTATGTACAGGAAAGAACTATATTTTTCAAAGATGACGGGAACTACAAGACACGCGC
GTTACGAAACGCTCTATGGGTCTAGTGTACTTTGTCGTACTGAAAAAGTTCTCACGGTACGGGCTTCCAATACATGTCCTTTCTTGATATAAAAAGTTTCTACTGCCCTTGATGTTCTGTGCGCG

eGFP

4,260 4,280 4,300 4,320 4,340 4,360

TGAAGTCAAGTTTGAAGGTGATACCCTTGTTAATAGAATCGAGTTAAAAGGTATTGATTTTAAAGAAGATGGAAACATTCTTGGACACAAATTGGAATACAACTATAACTCACACAATGTTTACA
ACTTCAGTTCAAACTTCCACTATGGGAACAATTATCTTAGCTCAATTTTCCATAACTAAAATTTCTTCTACCTTTGTAAGAACCTGTGTTTAACCTTATGTTGATATTGAGTGTGTTACAAATGT

eGFP

4,380 4,400 4,420 4,440 4,460 4,480 4,500

TCATGGCAGACAAACAAAAGAATGGAATCAAAGTAAACTTCAAAATTAGACACAACATTGAAGATGGAAGCGTTCAACTAGCAGACCATTATCAACAAAATACTCCAATTGGCGATGGCCCTGTC
AGTACCGTCTGTTTGTTTTCTTACCTTAGTTTCATTTGAAGTTTTAATCTGTGTTGTAACTTCTACCTTCGCAAGTTGATCGTCTGGTAATAGTTGTTTTATGAGGTTAACCGCTACCGGGACAG

eGFP

4,520 4,540 4,560 4,580 4,600 4,620

CTTTTACCAGACAACCATTACCTGTCCACACAATCTGCCCTATCGAAAGATCCCAACGAAAAGAGAGACCACATGGTCCTTCTTGAGTTTGTAACAGCAGCAGGGATTACACATGGCATGGATGA
GAAAATGGTCTGTTGGTAATGGACAGGTGTGTTAGACGGGATAGCTTTCTAGGGTTGCTTTTCTCTCTGGTGTACCAGGAAGAACTCAAACATTGTCGTCGTCCCTAATGTGTACCGTACCTACT

eGFP

4,640 4,660 4,680 4,700 4,720 4,740

ACTATACAAAGGCGCCTAAGGATCCTAGCTCGAGTTATATAATATATTTATGTACTCACAATGGGGTCTACAAAAAAAAAAAAAATAAAGATATATATATATATATATTATCTTTCATTTTATCAT
TGATATGTTTCCGCGGATTCCTAGGATCGAGCTCAATATATTATATAAATACATGAGTGTTACCCCAGATGTTTTTTTTTTTTTTATTTCTATATATATATATATATAATAGAAAGTAAAATAGTA

eGFP hsp86 3'

4,760 4,780 4,800 4,820 4,840 4,860

CACCCCAATATACATATATATATATTTTTATATTTTTATATATTTATATTTTTCTTTTATTTATCCATAAAAGAAAATTAAATATAAATTTATACCTTTTATTTGAATTACCATTTGTATAAAATAT
GTGGGGTTATATGTATATATATATAAAAATATAAAAATATATAAATATAAAAAGAAAATAAATAGGTATTTTCTTTTAATTTATATTTAAATATGGAAAATAAACTTAATGGTAAACATATTTTATA

hsp86 3'

4,880 4,900 4,920 4,940 4,960 4,980 5,000

AAAAAAAAAAAAAAATAATAAAAAGAAGAAAAAACGAAAAAAGTAATATTTAAATAATAAAGATCAATATACATAATAAAAAAAAAAAAAAAAAAAAAAAATTAAAATTTAAAAAATTAAACATTG
TTTTTTTTTTTTTTTATTATTTTTCTTCTTTTTTGCTTTTTTCATTATAAATTTATTATTTCTAGTTATATGTATTATTTTTTTTTTTTTTTTTTTTTTTTAATTTTAAATTTTTTAATTTGTAAC

hsp86 3'

5,020 5,040 5,060 5,080 5,100 5,120

AAATAATTTCATATACACACATATACATACATACATATATATATATATATATATATATTTATACATTTATGCATATAAAATATATTAACAACTTATGTGTATTCATATATATTTTTTTACATATG
TTTATTAAAGTATATGTGTGTATATGTATGTATGTATATATATATATATATATATATAAATATGTAAATACGTATATTTTATATAATTGTTGAATACACATAAGTATATATAAAAAAATGTATAC

hsp86 3'

5,140 5,160 5,180 5,200 5,220 5,240

TATTGAAAGACTTTACTGAGACATGAAATATCTAATAACCAAACGTTTATAAATTCTAATTATATGTAAAGCATAATATAGCTTTTGTCTAATTTAATTCCTTAAAACGTATGTATTAAAAATAT
ATAACTTTCTGAAATGACTCTGTACTTTATAGATTATTGGTTTGCAAATATTTAAGATTAATATACATTTCGTATTATATCGAAAACAGATTAAATTAAGGAATTTTGCATACATAATTTTTATA

hsp86 3'

5,260 5,280 5,300 5,320 5,340 5,360

AGTAAGATGGGGAAATTATAATTTTTTTTTTAAAGGTTTTTCTTTGATACAGTAAAAAAAAAAAAATAAAAAAAAATAATAATAATAAAAAAATAAAAAAATAAAAAAAAATAAAATAATTATAAA
TCATTCTACCCCTTTAATATTAAAAAAAAAATTTCCAAAAAGAAACTATGTCATTTTTTTTTTTTTATTTTTTTTTATTATTATTATTTTTTTATTTTTTTATTTTTTTTTATTTTATTAATATTT

hsp86 3'

5,380 5,400 5,420 5,440 5,460 5,480 5,500

GATATATATAATTCCTCCCAAATACAAATGATTAAGTTTAAAAATTAATAAATAAATCAAAAAAAAATTTAATAATAATTGTATTATTTTAAGTGTAGTTAATTCATCAAATAGCATGCCTGCAG
CTATATATATTAAGGAGGGTTTATGTTTACTAATTCAAATTTTTAATTATTTATTTAGTTTTTTTTTAAATTATTATTAACATAATAAAATTCACATCAATTAAGTAGTTTATCGTACGGACGTC

hsp86 3'

5,520 5,540 5,560 5,580 5,600 5,620

```
GTCGACagtcTCCGGActcgGCTAGCcgatTACAACCATTTTGTAAAAAAAATTAAAATATATTTATATAATATTATTTTATTTTATTATATATTATATTATTTTTATTTTTATTTTTTATTTTTT
CAGCTGtcagAGGCCTgagcCGATCGgctaATGTTGGTAAAACATTTTTTTTAATTTTATATAAATATATTATAATAAAATAAAATAATATATAATATAATAAAAATAAAAATAAAAAATAAAAAA
```

5,640 5,660 5,680 5,700 5,720 5,740

```
TTTCTCTACAAATTTTATCTATTGGTTTATTATAAAAATATCTATTTCTAATAATAAATAATTAAGATATCAAGCGGCCTTCCGCGAATAACTTCGTATAGGATACTTTATACGAAGTTATtata
AAAGAGATGTTTAAAATAGATAACCAAATAATATTTTTATAGATAAAGATTATTATTTATTAATTCTATAGTTCGCCGGAAGGCGCTTATTGAAGCATATCCTATGAAATATGCTTCAATAatat
```

lox2272

5,760 5,780 5,800 5,820 5,840 5,860

```
tatgtatatatatatatatttatatatttttatattcttttagGGTCACCTAGGATGAAGGTGAGCAAGGGCGAGGAGGATAACATGGccatcatcaaggagttcatgcgcttcaaggtgcacatg
atacatatatatatatataaatatataaaatataagaaaatcCCAGTGGATCCTACTTCCACTCGTTCCCGCTCCTCCTATTGTACCggtagtagttcctcaagtacgcgaagttccacgtgtac
```

SERA2 intron | mCherry

5,880 5,900 5,920 5,940 5,960 5,980 6,000

```
gagggctccgtgaacggccacgagttcgagatcgagggcgagggcgagggccgcccctacgagggcacccagaccgccaagctgaaggtgaccaagggtggccccctgcccttcgcctgggacat
ctcccgaggcacttgccggtgctcaagctctagctcccgctcccgctcccggcggggatgctcccgtgggtctggcggttcgacttccactggttcccaccgggggacgggaagcggaccctgta
```

mCherry

6,020 6,040 6,060 6,080 6,100 6,120

```
cctgtcccctcagttcatgtacggctccaaggcctacgtgaagcaccccgccgacatccccgactacttgaagctgtccttccccgagggcttcaagtgggagcgcgtgatgaacttcgaggacg
ggacaggggagtcaagtacatgccgaggttccggatgcacttcgtggggcggctgtaggggctgatgaacttcgacaggaaggggctcccgaagttcaccctcgcgcactacttgaagctcctgc
```

mCherry

6,140 6,160 6,180 6,200 6,220 6,240

```
gcggcgtggtgaccgtgacccaggactcctccctgcaggacggcgagttcatctacaaggtgaagctgcgcggcaccaacttcccctccgacggccccgtaatgcagaagaagaccatgggctgg
cgccgcaccactggcactgggtcctgaggagggacgtcctgccgctcaagtagatgttccacttcgacgcgccgtggttgaaggggaggctgccggggcattacgtcttcttctggtacccgacc
```

mCherry

6,260 6,280 6,300 6,320 6,340 6,360

```
gaggcctcctccgagcggatgtaccccgaggacggcgccctgaagggcgagatcaagcagaggctgaagctgaaggacggcggccactacgacgctgaggtcaagaccacctacaaggccaagaa
ctccggaggaggctcgcctacatggggctcctgccgcgggacttcccgctctagttcgtctccgacttcgacttcctgccgccggtgatgctgcgactccagttctggtggatgttccggttctt
```

mCherry

6,380 6,400 6,420 6,440 6,460 6,480 6,500

```
gcccgtgcagctgcccggcgcctacaacgtcaacatcaagttggacatcacctcccacaacgaggactacaccatcgtggaacagtacgaacgcgccgagggccgccactccaccggcggcatgg
cgggcacgtcgacgggccgcggatgttgcagttgtagttcaacctgtagtggagggtgttgctcctgatgtggtagcaccttgtcatgcttgcgcggctcccggcggtgaggtggccgccgtacc
```

mCherry

6,520 6,540 6,560 6,580 6,600 6,620

```
acgagctgtacaagtaacGATAAATAAATAAATAAAATATATATATATATGTAGAGATATATATATACTTAATTAGGGCGATATGATAATTGAAAGGATTATTATTTTTTAATTTTTTTAGTATA
tgctcgacatgttcattgCTATTTATTTATTTATTTTATATATATATATACATCTCTATATATATATGAATTAATCCCGCTATACTATTAACTTTCCTAATAATAAAAAATTAAAAAAATCATAT
```

mCherry | Pfpkg 3'UTR

6,640 6,660 6,680 6,700 6,720 6,740

TTTTTAGAAATCATATATATTCATAATAATATATTTGTTCCATCATATATATTATATATATATATATATTATATATATATATAATAATTATAAATTTTTGAAAAATGTTATTTTTATTATTAATA
AAAAATCTTTAGTATATATAAGTATTATTATATAAACAAGGTAGTATATATAATATATATATATATATAATATATATATATATTATTAATATTTAAAAACTTTTTACAATAAAAATAATAATTAT

Pfpkg 3'UTR

6,760 6,780 6,800 6,820 6,840 6,860

ATTTTTTTTTTTTTTTTCATTTGTTTGTACAATATAATGAATTTATATCTTGCGGTGTTTTTTTTTATTCTCTCCTTTTTTATATTATATTTATTTTATTTTATTTTATTCTTTTTCTTTTTTTTTTTT
TAAAAAAAAAAAAAAAAGTAAACAAACATGTTATATTACTTAAATATAGAACGCCACAAAAAAAAATAAGAGAGGAAAAAATATAATATAAAATAAAATAAAATAAGAAAAAGAAAAAAAAAAAA

Pfpkg 3'UTR

6,880 6,900 6,920 6,940 6,960 6,980 7,000

TCTTTTTTTTTTTTTGTTTGTTTTTAATTTTACAAATATATTTAAATAAAAATTCAAATGGTTCTAAATTTTTTTAATAAATAAATAAAGTAATATTTACTATTTTAATTAGTGTTCAAAATGAA
AGAAAAAAAAAAAAACAAACAAAAATTAAAATGTTTATATAAATTTATTTTTAAGTTTACCAAGATTTAAAAAAATTATTTATTTATTTCATTATAAATGATAAAATTAATCACAAGTTTTACTT

Pfpkg 3'UTR

7,020 7,040 7,060 7,080 7,100 7,120

GAAGAATCAAAAAAAATGAAAATATGAAATTTGTATAAATATTTATAGGTATTTATATATATATATATATATATATATATATATATACACGGTGTTAATGCTTTACATTTTTTTTTTTTTTTTTTCCT
CTTCTTAGTTTTTTTTACTTTTATACTTTAAACATATTTATAAATATCCATAAATATATATATATATATATATATATATATATATATGTGCCACAATTACGAAATGTAAAAAAAAAAAAAAAAAAGGA

Pfpkg 3'UTR

7,140 7,160 7,180 7,200 7,220 7,240

ATGTCTATATATTTATTAATCCTTATATTTTTATATAAATTGTGCATT
TACAGATATATAAATAATTAGGAATATAAAAATATATTTAACACGTAA

Pfpkg 3'UTR

7,260 7,270 7,280 7,290

pDC_loxNmCherry_lox22PKG (7105 bp)

TATTTTCTAACAGAATTAGTAACAGGTGGAGAATTATATGATGCTATTAGAAAATTAGGTTTATTATCTAAATCACAAGCTCAATTTTATTTAGGTTCTATCATTTTAGCTATTGAATATTTACA
ATAAAAGATTGTCTTAATCATTGTCCACCTCTTAATATACTACGATAATCTTTTAATCCAAATAATAGATTTAGTGTTCGAGTTAAAATAAATCCAAGATAGTAAAATCGATAACTTATAAATGT

5' homology arm

20 40 60 80 100 120

TGAAAGAAATATTGTATATAGAGATTTAAAACCAGAAAACATTTTATTAGATAAACAAGGTTATGTAAAACTAATCGATTTTGGTTGTGCCAAAAAGGTACAAGGTAGAGCTTATACATTAGTAG
ACTTTCTTTATAACATATATCTCTAAATTTTGGTCTTTTGTAAAATAATCTATTTGTTCCAATACATTTTGATTAGCTAAAACCAACACGGTTTTTCCATGTTCCATCTCGAATATGTAATCATC

5' homology arm

140 160 180 200 220 240

GTACACCTCATTATATGGCACCTGAGGTTATTTTAGGAAAAGGTTATGGATGTACTGTTGACATATGGGCATTGGGAATATGCCTATATGAATTTATATGTGGTCCATTACCATTTGGTAATGAT
CATGTGGAGTAATATACCGTGGACTCCAATAAAATCCTTTTCCAATACCTACATGACAACTGTATACCCGTAACCCTTATACGGATATACTTAAATATACACCAGGTAATGGTAAACCATTACTA

5' homology arm

260 280 300 320 340 360

GAAGAAGATCAATTAGAAATTTTCagaGATATTCTGACAGGCCAGCTGACCTTTCCGGATTATGTTACCGATACCGATAGCATCAACCTGATGAAACGCCTGCTGTGTCGTCTGCCGCAGGGTCG
CTTCTTCTAGTTAATCTTTAAAAGtctCTATAAGACTGTCCGGTCGACTGGAAAGGCCTAATACAATGGCTATGGCTATCGTAGTTGGACTACTTTGCGGACGACACAGCAGACGGCGTCCCAGC

5' homology arm | pfpkgsynth

380 400 420 440 460 480 500

TATTGGTTGTAGCATTAATGGTTTTAAAGACATCAAAGATCACCCGTTCTTCAGCAACTTTAACTGGGATAAACTGGCAGGTCGTCTGCTGGACCCTCCGCTGGTTAGCAAAAGCGAAACCTATG
ATAACCAACATCGTAATTACCAAAATTTCTGTAGTTTCTAGTGGGCAAGAAGTCGTTGAAATTGACCCTATTTGACCGTCCAGCAGACGACCTGGGAGGCGACCAATCGTTTTCGCTTTGGATAC

pfpkgsynth

520 540 560 580 600 620

CAGAGGATATCGACATCAAACAAATCGAAGAAGAGGACGCAGAGGACGATGAGGAACCGCTGAATGACGAAGATAATTGGGATATCGATTTTTAAgaggttaccatcgagggatatggcagctta
GTCTCCTATAGCTGTAGTTTGTTTAGCTTCTTCTCCTGCGTCTCCTGCTACTCCTTGGCGACTTACTGCTTCTATTAACCCTATAGCTAAAAATTctccaatggtagctccctataccgtcgaat

pfpkgsynth | Pb DT 3'UTR

640 660 680 700 720 740

atgttcgtttttcttatttatatatttataccaattgattgtatttataactgtaaaaatgtgtatgttgtgtgcatattttttttgtgcatgcacatgcatgtaaatagctaaaattatgaac
tacaagcaaaaagaataaatatataaatatggttaactaacataaatattgacatttttacacatacaacacacgtataaaaaaaacacgtacgtgtacgtacatttatcgattttaatacttg

Pb DT 3'UTR

760 780 800 820 840 860

atttatttttgttcagaaaaaaaaaactttacacacataaaatggctagtatgaatagccatattttatataaattaaatcctatgaatttatgaccatattaaaaatttagatatttatgga
taaaataaaaacaagtcttttttttttgaaatgtgtgtattttaccgatcatacttatcggtataaaatatatttaatttaggatacttaaatactggtataatttttaaatctataaatacct

Pb DT 3'UTR

880 900 920 940 960 980 1,000

acataatatgtttgaaacaataagacaaaattattattattattattatttttactgttataattatgtgtctccttcaatgattcataaatagttggacttgattttaaaatgtttataatat
tgtattatacaaactttgttattctgttttaataataataataataataaaaatgacaatattaatacacagaggaagttactaagtatttatcaacctgaactaaaattttacaaatattata

Pb DT 3'UTR

1,020 1,040 1,060 1,080 1,100 1,120

gattagcatagttaaataaaaaaagttgaaaaattaaaaaaaaacatataaacacaaatgatggtttttccttcaatttcgatatcatatagcaatcgtattagcaggtaaatgagcaaggttaa
ctaatcgtatcaatttatttttttcaactttttaatttttttttgtatatttgtgtttactaccaaaaaggaagttaaagctatagtatatcgttagcataatcgtccatttactcgttccaatt

Pb DT 3'UTR

1,140 1,160 1,180 1,200 1,220 1,240

taaaaaaatggtaaaaaaaaaaaaattggggaaaaataaacaaggtgtctttatctacactttggctttacaatgtataatttatatgcatacaaaaaaattttgataaaattgtaaaaaggtgaa
atttttttaccatttttttttttttaaccccttttttatttgttccacagaaatagatgtgaaaccgaaatgttacatattaaatatacgtatgtttttttaaaactattttaacatttttccactt

Pb DT 3'UTR

1,260 1,280 1,300 1,320 1,340 1,360

taaaataacattgtaacagtagtagatagtaaagggaaggtgttgctcaaatagtgtcgaaacaaaactggcataaagaaaaattgatattgagcagaggatatgcgcataatggtattttgttt
attttattgtaacattgtcatcatctatcatttcccttccacaacgagtttatcacagctttgttttgaccgtatttctttttaactataactcgtctcctatacgcgtattaccataaaacaaa

Pb DT 3'UTR

1,380 1,400 1,420 1,440 1,460 1,480 1,500

gtttgtttgttttctcatttttttgagacagctcaattctttatgtccacaacatcatcggacttttcttcttcagggtaggcGGCCGCTCGAGCATGCATCTAGATTTTATATAATAAAAAATGG
caaacaaacaaaagagtaaaaaaactctgtcgagttaagaaatacaggtgttgtagtagcctgaaaagaagaagtcccatccgCCGGCGAGCTCGTACGTAGATCTAAAATATATTATTTTTTACC

Pb DT 3'UTR

1,520 1,540 1,560 1,580 1,600 1,620

AGAAGTAGAAATAGTAAAAAATAAAAAAAGGTTACgtgcggttaacaATAACTTCGTATAGTATACCTTATACGAAGTTATtatatatgtatatatatatatatttatatattttatattctttt
TCTTCATCTTTATCATTTTTTATTTTTTTCCAATGcacgccaattgtTATTGAAGCATATCATATGGAATATGCTTCAATAatatatacatatatatatatataaatatataaaatataagaaaa

loxN site SERA2 intron

1,640 1,660 1,680 1,700 1,720 1,740

agAAAAAATGAaggtgagcaagggcgaggaggataacatggccatcatcaaggagttcatgcgcttcaaggtgcacatggagggctccgtgaacggccacgagttcgagatcgagggcgagggcg
tcTTTTTTACTtccactcgttcccgctcctcctattgtaccggtagtagttcctcaagtacgcgaagttccacgtgtacctcccgaggcacttgccggtgctcaagctctagctcccgctcccgc

mCherry

1,760 1,780 1,800 1,820 1,840 1,860

agggccgcccctacgagggcacccagaccgccaagctgaaggtgaccaagggtggcccccctgcccttcgcctgggacatcctgtcccctcagttcatgtacggctccaaggcctacgtgaagcac
tcccggcggggatgctcccgtgggtctggcggttcgacttccactggttcccaccggggggacgggaagcggaccctgtaggacaggggagtcaagtacatgccgaggttccggatgcacttcgtg

mCherry

1,880 1,900 1,920 1,940 1,960 1,980 2,000

cccgccgacatccccgactacttgaagctgtccttccccgagggcttcaagtgggagcgcgtgatgaacttcgaggacggcggcgtggtgaccgtgacccaggactcctccctgcaggacggcga
gggcggctgtaggggctgatgaacttcgacaggaaggggctcccgaagttcaccctcgcgcactacttgaagctcctgccgccgcaccactggcactgggtcctgaggagggacgtcctgccgct

mCherry

2,020 2,040 2,060 2,080 2,100 2,120

gttcatctacaaggtgaagctgcgcggcaccaacttcccctccgacggccccgtaatgcagaagaagatcatgggctgggaggcctcctccgagcggatgtaccccgaggacggcgccctgaagg
caagtagatgttccacttcgacgcgccgtggttgaaggggaggctgccggggcattacgtcttcttctagtacccgaccctccggaggaggctcgcctacatggggctcctgccgcgggacttcc

mCherry

2,140 2,160 2,180 2,200 2,220 2,240

```
gcgagatcaagcagaggctgaagctgaaggacggcggccactacgacgctgaggtcaagaccacctacaaggccaagaagcccgtgcagctgcccggcgcctacaacgtcaacatcaagttggac
cgctctagttcgtctccgacttcgacttcctgccgccggtgatgctgcgactccagttctggtggatgttccggttcttcgggcacgtcgacgggccgcggatgttgcagttgtagttcaacctg
```
mCherry

2,260 2,280 2,300 2,320 2,340 2,360

```
atcacctcccacaacgaggactacaccatcgtggaacagtacgaacgcgccgagggccgccactccaccggcggcatggacgagctgtacaagtaacTCGAGTTATATAATATATTTATGTACTC
tagtggagggtgttgctcctgatgtggtagcaccttgtcatgcttgcgcggctcccggcggtgaggtggccgccgtacctgctcgacatgttcattgAGCTCAATATATTATATAAATACATGAG
```
mCherry hsp86 3'

2,380 2,400 2,420 2,440 2,460 2,480 2,500

```
ACAATGGGGTCTACAAAAAAAAAAAAAATAAAGATATATATATATATATATTATCTTTCATTTTATCATCACCCCAATATACATATATATATTTTTATATTTTTATATATTTATATTTTTCTTTTA
TGTTACCCCAGATGTTTTTTTTTTTTTTATTTCTATATATATATATATATAATAGAAAGTAAAATAGTAGTGGGGTTATATGTATATATATAAAAATATAAAAATATATAAATATAAAAAGAAAAT
```
hsp86 3'

2,520 2,540 2,560 2,580 2,600 2,620

```
TTTATCCATAAAAGAAAATTAAATATAAATTTATACCTTTTATTTGAATTACCATTTGTATAAAATATAAAAAAAAAAAAAAAATAATAAAAAGAAGAAAAAACGAAAAAAGTAATATTTAAATAA
AAATAGGTATTTTCTTTTAATTTATATTTAAATATGGAAAATAAACTTAATGGTAAACATATTTTATATTTTTTTTTTTTTTTTATTATTTTTCTTCTTTTTTGCTTTTTTCATTATAAATTTATT
```
hsp86 3'

2,640 2,660 2,680 2,700 2,720 2,740

```
TAAAGATCAATATACATAATAAAAAAAAAAAAAAAAAAAAAAAAATTAAAATTTAAAAAATTAAACATTGAAATAATTTCATATACACACATATACATACATACATATATATATATATATATATATA
ATTTCTAGTTATATGTATTATTTTTTTTTTTTTTTTTTTTTTTTTTAATTTTAAATTTTTTAATTTGTAACTTTATTAAAGTATATGTGTGTATATGTATGTATGTATATATATATATATATATAT
```
hsp86 3'

2,760 2,780 2,800 2,820 2,840 2,860

```
TTTATACATTTATGCATATAAAATATATTAACAACTTATGTGTATTCATATATATTTTTTTACATATGTATTGAAAGACTTTACTGAGACATGAAATATCTAATAACCAAACGTTTATAAATTCT
AAATATGTAAATACGTATATTTTATATAATTGTTGAATACACATAAGTATATATAAAAAAATGTATACATAACTTTCTGAAATGACTCTGTACTTTATAGATTATTGGTTTGCAAATATTTAAGA
```
hsp86 3'

2,880 2,900 2,920 2,940 2,960 2,980 3,000

```
AATTATATGTAAAGCATAATATAGCTTTTGTCTAATTTAATTCCTTAAAACGTATGTATTAAAAATATAGTAAGATGGGGAAATTATAATTTTTTTTTTAAAGGTTTTTCTTTGATACAGTAAAA
TTAATATACATTTCGTATTATATCGAAAACAGATTAAATTAAGGAATTTTGCATACATAATTTTTATATCATTCTACCCCTTTAATATTAAAAAAAAAATTTCCAAAAAGAAACTATGTCATTTT
```
hsp86 3'

3,020 3,040 3,060 3,080 3,100 3,120

```
AAAAAAAATAAAAAAAAAATAATAATAATAAAAAAAATAAAAAAAATAAAAAAAAAATAAAATAATTATAAAGATATATATAATTCCTCCCAAATACAAATGATTAAGTTTAAAAATTAATAAATAAAT
TTTTTTTTATTTTTTTTTTATTATTATTATTTTTTTTATTTTTTTTATTTTTTTTTTATTTTATTAATATTTCTATATATATTAAGGAGGGTTTATGTTTACTAATTCAAATTTTTAATTATTTATTTA
```
hsp86 3'

3,140 3,160 3,180 3,200 3,220 3,240

```
CAAAAAAAAATTTAATAATAATTGTATTATTTTAAGTGTAGTTAATTCATCAAATAGCATGCCTGCAGGTCGACagtGGCCTTCCGCGAATAACTTCGTATAGGATACTTTATACGAAGTTATta
GTTTTTTTTTAAATTATTATTAACATAATAAAATTCACATCAATTAAGTAGTTTATCGTACGGACGTCCAGCTGtcaCCGGAAGGCGCTTATTGAAGCATATCCTATGAAATATGCTTCAATAat
```
hsp86 3' lox2272

3,260 3,280 3,300 3,320 3,340 3,360

```
tatatgtatatatatatatatttatatatttttatattcttttagGCAGCTATTTCTTCATTATTAATAGCGGCAAATTTGATGTGTACGTGAACGACAAAAAAGTGAAAACCATGGGTAAAGGTA
atatacatatatatatatataaatatataaaaatataagaaaatcCGTCGATAAAGAAGTAATAATTATCGCCGTTTAAACTACACATGCACTTGCTGTTTTTTCACTTTTGGTACCCATTTCCAT
SERA2 intron | PKGsynth_cDNA
3,380  3,400  3,420  3,440  3,460  3,480  3,500

GCAGCTTTGGTGAAGCAGCACTGATTCATAATACCCAGCGTAGCGCAACCATTATTGCAGAAACCGATGGCACCCTGTGGGGTGTTCAGCGTAGCACCTTTCGTGCAACCCTGAAACAGCTGAGC
CGTCGAAACCACTTCGTCGTGACTAAGTATTATGGGTCGCATCGCGTTGGTAATAACGTCTTTGGCTACCGTGGGACACCCCACAAGTCGCATCGTGGAAAGCACGTTGGGACTTTGTCGACTCG
PKGsynth_cDNA
3,520  3,540  3,560  3,580  3,600  3,620

AATCGTAACTTTAATGAAAACCGCACCTTTATCGATAGCGTGAGCGTTTTTGATATGCTGACCGAAGCACAGAAAAACATGATTACCAATGCCTGCGTGATCCAGAACTTTAAAAGTGGTGAAgC
TTAGCATTGAAATTACTTTTGGCGTGGAAATAGCTATCGCACTCGCAAAAACTATACGACTGGCTTCGTGTCTTTTTGTACTAATGGTTACGGACGCACTAGGTCTTGAAATTTTCACCACTTcG
PKGsynth_cDNA
3,640  3,660  3,680  3,700  3,720  3,740

CATTGTGAAACAGGGCGATTATGGTGATGTTCTGgcTATTCTGAAAGAGGGTAAAGCCACCGTCTATATCAATGATGAAGAGATTCGTGTGCTGGAAAAAGGCTCATATTTTGGTGAACGTGCAC
GTAACACTTTGTCCCGCTAATACCACTACAAGACcgATAAGACTTTCTCCCATTTCGGTGGCAGATATAGTTACTACTTCTCTAAGCACACGACCTTTTTCCGAGTATAAAACCACTTGCACGTG
PKGsynth_cDNA
3,760  3,780  3,800  3,820  3,840  3,860

TGCTGTATGATGAACCGCGTTCAGCCACAATTATTGCAAAAGAACCGACCGCATGTGCAAGCATTTGTCGTAAACTGCTGAATATTGTTCTGGGCAATCTGCAGGTTGTTCTGTTTCGCAATATT
ACGACATACTACTTGGCGCAAGTCGGTGTTAATAACGTTTTCTTGGCTGGCGTACACGTTCGTAAACAGCATTTGACGACTTATAACAAGACCCGTTAGACGTCCAACAAGACAAAGCGTTATAA
PKGsynth_cDNA
3,880  3,900  3,920  3,940  3,960  3,980  4,000

ATGACAGAAGCACTGCAGCAGAGCGAAATCTTTAAACAGTTTAGCGGTGATCAGCTGAATGATCTGGCAGATACCGCAATTGTTCGTGATTATCCGGCAAACTATAACATCCTGCACAAAGATAA
TACTGTCTTCGTGACGTCGTCTCGCTTTAGAAATTTGTCAAATCGCCACTAGTCGACTTACTAGACCGTCTATGGCGTTAACAAGCACTAATAGGCCGTTTGATATTGTAGGACGTGTTTCTATT
PKGsynth_cDNA
4,020  4,040  4,060  4,080  4,100  4,120

AGTTAAAAGCGTGAAATACATCATCGTGCTGGAAGGTAAAGTTGAACTGTTTCTGGATGATACCAGCATTGGTATTCTGAGCCGTGGTATGAGTTTTGGTGATCAGTATGTTCTGAATCAGAAAC
TCAATTTTCGCACTTTATGTAGTAGCACGACCTTCCATTTCAACTTGACAAAGACCTACTATGGTCGTAACCATAAGACTCGGCACCATACTCAAAACCACTAGTCATACAAGACTTAGTCTTTG
PKGsynth_cDNA
4,140  4,160  4,180  4,200  4,220  4,240

AGCCGTTCAAACACACCATTAAAAGCCTGGAAGTTTGCAAAATTGCCCTGATTACCGAAACCTGTCTGGCCGATTGTCTGGGTAATAACAATATTGATGCCAGCATCGATTACAATAACAAAAAA
TCGGCAAGTTTGTGTGGTAATTTTCGGACCTTCAAACGTTTTAACGGGACTAATGGCTTTGGACAGACCGGCTAACAGACCCATTATTGTTATAACTACGGTCGTAGCTAATGTTATTGTTTTTT
PKGsynth_cDNA
4,260  4,280  4,300  4,320  4,340  4,360

TCCATCATCAAAAAAATGTACATCTTTCGCTATCTGACCGATAAACAGTGCAATCTGCTGATTGAAGCATTTCGTACCACCCGTTATGAAGAAGGCGATTATATCATTCAAGAAGGTGAAGTTGG
AGGTAGTAGTTTTTTTACATGTAGAAAGCGATAGACTGGCTATTTGTCACGTTAGACGACTAACTTCGTAAAGCATGGTGGGCAATACTTCTTCCGCTAATATAGTAAGTTCTTCCACTTCAACC
PKGsynth_cDNA
4,380  4,400  4,420  4,440  4,460  4,480  4,500
```

CAGCCGCTTCTACATTATCAAAAATGGTGAAGTCGAAATCGTCAAAAACAAAAAACGTCTGCGTACCCTGGGCAAAAATGATTATTTTGGCGAGCGTGCCCTGCTGTACGACGAGCCTCGTACCG
GTCGGCGAAGATGTAATAGTTTTTACCACTTCAGCTTTAGCAGTTTTTGTTTTTTGCAGACGCATGGGACCCGTTTTTACTAATAAAACCGCTCGCACGGGACGACATGCTGCTCGGAGCATGGC

PKGsynth_cDNA

4,520 4,540 4,560 4,580 4,600 4,620

CAAGCGTTATTAGCAAAGTTAATAATGTGGAATGCTGGTTTGTGGACAAAAGCGTTTTTCTGCAGATTATTCAGGGTCCTATGCTGGCACATCTGGAAGAACGCATTAAAATGCAGGATACCAAA
GTTCGCAATAATCGTTTCAATTATTACACCTTACGACCAAACACCTGTTTTCGCAAAAAGACGTCTAATAAGTCCCAGGATACGACCGTGTAGACCTTCTTGCGTAATTTTACGTCCTATGGTTT

PKGsynth_cDNA

4,640 4,660 4,680 4,700 4,720 4,740

GTGGAAATGGATGAACTGGAAACCGAACGTATTATTGGTCGTGGCACCTTTGGCACCGTTAAACTGGTGCATCACAAACCGACCAAAATTCGTTATGCACTGAAATGCGTTAGCAAACGCgccAT
CACCTTTACCTACTTGACCTTTGGCTTGCATAATAACCAGCACCGTGGAAACCGTGGCAATTTGACCACGTAGTGTTTGGCTGGTTTTAAGCAATACGTGACTTTACGCAATCGTTTGCGcggTA

PKGsynth_cDNA

4,760 4,780 4,800 4,820 4,840 4,860

TATCAATCTGAACCAGCAGAACAACATTAAACTGGAACGTGAAATTACCGCAGAAAACGATCATCCGTTTATTATCCGTCTGGTGCGTACCTTTAAAGATAGCAAATATTTCTACTTCCTGACCG
ATAGTTAGACTTGGTCGTCTTGTTGTAATTTGACCTTGCACTTTAATGGCGTCTTTTGCTAGTAGGCAAATAATAGGCAGACCACGCATGGAAATTTCTATCGTTTATAAAGATGAAGGACTGGC

PKGsynth_cDNA

4,880 4,900 4,920 4,940 4,960 4,980 5,000

AACTGGTTACCGGTGGTGAACTGTATGACGCAATTCGCAAACTGGGTCTGCTGAGCAAAAGCCAGGCACAGTTTTATCTGGGTAGCATTATTCTGGCCATCGAATATCTGCATGAACGCAATATT
TTGACCAATGGCCACCACTTGACATACTGCGTTAAGCGTTTGACCCAGACGACTCGTTTTCGGTCCGTGTCAAAATAGACCCATCGTAATAAGACCGGTAGCTTATAGACGTACTTGCGTTATAA

PKGsynth_cDNA

5,020 5,040 5,060 5,080 5,100 5,120

GTGTATCGTGATCTGAAACCGGAAAACATTCTGCTGGATAAACAGGGCTATGTGAAACTGATTGATTTTGGGTGTGCCAAAAAAGTTCAGGGTCGTGCAgcTgCCCTGGTTGGTACACCGCATTA
CACATAGCACTAGACTTTGGCCTTTTGTAAGACGACCTATTTGTCCCGATACACTTTGACTAACTAAAACCCACACGGTTTTTTCAAGTCCCAGCACGTcgAcGGGACCAACCATGTGGCGTAAT

PKGsynth_cDNA

5,140 5,160 5,180 5,200 5,220 5,240

TATGGCACCGGAAGTTATTCTGGGAAAAGGTTATGGTTGTACCGTTGATATTTGGGCACTGGGTATTTGTCTGTACGAATTTATCTGTGGTCCGCTGCCGTTTGGTAACGATGAAGAGGATCAGC
ATACCGTGGCCTTCAATAAGACCCTTTTCCAATACCAACATGGCAACTATAAACCCGTGACCCATAAACAGACATGCTTAAATAGACACCAGGCGACGGCAAACCATTGCTACTTCTCCTAGTCG

PKGsynth_cDNA

5,260 5,280 5,300 5,320 5,340 5,360

TGGAAATTTTTCGTGATATTCTGACAGGCCAGCTGACCTTTCCGGATTATGTTACCGATACCGATAGCATCAACCTGATGAAACGCCTGCTGTGTCGTCTGCCGCAGGGTCGTATTGGTTGTAGC
ACCTTTAAAAAGCACTATAAGACTGTCCGGTCGACTGGAAAGGCCTAATACAATGGCTATGGCTATCGTAGTTGGACTACTTTGCGGACGACACAGCAGACGGCGTCCCAGCATAACCAACATCG

PKGsynth_cDNA

5,380 5,400 5,420 5,440 5,460 5,480 5,500

ATTAATGGTTTTAAAGACATCAAAGATCACCCGTTCTTCAGCAACTTTAACTGGGATAAACTGGCAGGTCGTCTGCTGGACCCTCCGCTGGTTgcCAAAgcCGAAACCTATGCAGAGGATATCGA
TAATTACCAAAATTTCTGTAGTTTCTAGTGGGCAAGAAGTCGTTGAAATTGACCCTATTTGACCGTCCAGCAGACGACCTGGGAGGCGACCAAcgGTTTcgGCTTTGGATACGTCTCCTATAGCT

PKGsynth_cDNA

5,520 5,540 5,560 5,580 5,600 5,620

```
CATCAAACAAATCGAAGAAGAGGACGCAGAGGACGATGAGGAACCGCTGAATGACGAAGATAATTGGGATATCGATTTTccTAgGTCTGTGAGTAAAGGAGAAGAACTTTTCACTGGAGTTGTCC
GTAGTTTGTTTAGCTTCTTCTCCTGCGTCTCCTGCTACTCCTTGGCGACTTACTGCTTCTATTAACCCTATAGCTAAAAggATcCAGACACTCATTTCCTCTTCTTGAAAAGTGACCTCAACAGG
```
PKGsynth_cDNA | eGFP

5,640 5,660 5,680 5,700 5,720 5,740

```
CAATTCTTGTTGAATTAGATGGTGATGTTAATGGGCACAAATTTTCTGTCAGTGGAGAGGGTGAAGGTGATGCAACATACGGAAAACTTACCCTTAAATTTATTTGCACTACTGGAAAACTACCT
GTTAAGAACAACTTAATCTACCACTACAATTACCCGTGTTTAAAAGACAGTCACCTCTCCCACTTCCACTACGTTGTATGCCTTTTGAATGGGAATTTAAATAAACGTGATGACCTTTTGATGGA
```
eGFP

5,760 5,780 5,800 5,820 5,840 5,860

```
GTTCCCTGGCCAACACTTGTCACTACTTTCGGTTATGGTGTTCAATGCTTTGCGAGATACCCAGATCACATGAAACAGCATGACTTTTTCAAGAGTGCCATGCCCGAAGGTTATGTACAGGAAAG
CAAGGGACCGGTTGTGAACAGTGATGAAAGCCAATACCACAAGTTACGAAACGCTCTATGGGTCTAGTGTACTTTGTCGTACTGAAAAAGTTCTCACGGTACGGGCTTCCAATACATGTCCTTTC
```
eGFP

5,880 5,900 5,920 5,940 5,960 5,980 6,000

```
AACTATATTTTTCAAAGATGACGGGAACTACAAGACACGCGCTGAAGTCAAGTTTGAAGGTGATACCCTTGTTAATAGAATCGAGTTAAAAGGTATTGATTTTAAAGAAGATGGAAACATTCTTG
TTGATATAAAAAGTTTCTACTGCCCTTGATGTTCTGTGCGCGACTTCAGTTCAAACTTCCACTATGGGAACAATTATCTTAGCTCAATTTTCCATAACTAAAATTTCTTCTACCTTTGTAAGAAC
```
eGFP

6,020 6,040 6,060 6,080 6,100 6,120

```
GACACAAATTGGAATACAACTATAACTCACACAATGTTTACATCATGGCAGACAAACAAAAGAATGGAATCAAAGTAAACTTCAAAATTAGACACAACATTGAAGATGGAAGCGTTCAACTAGCA
CTGTGTTTAACCTTATGTTGATATTGAGTGTGTTACAAATGTAGTACCGTCTGTTTGTTTTCTTACCTTAGTTTCATTTGAAGTTTTAATCTGTGTTGTAACTTCTACCTTCGCAAGTTGATCGT
```
eGFP

6,140 6,160 6,180 6,200 6,220 6,240

```
GACCATTATCAACAAAATACTCCAATTGGCGATGGCCCTGTCCTTTTACCAGACAACCATTACCTGTCCACACAATCTGCCCTATCGAAAGATCCCAACGAAAAGAGAGACCACATGGTCCTTCT
CTGGTAATAGTTGTTTTATGAGGTTAACCGCTACCGGGACAGGAAAATGGTCTGTTGGTAATGGACAGGTGTGTTAGACGGGATAGCTTTCTAGGGTTGCTTTTCTCTCTGGTGTACCAGGAAGA
```
eGFP

6,260 6,280 6,300 6,320 6,340 6,360

```
TGAGTTTGTAACAGCAGCAGGGATTACACATGGCATGGATGAACTATACAAAGGCGCCTAAGGATCCTAGCTCGAGATAAATAAATAAATAAAATATATATATATATGTAGAGATATATATATAC
ACTCAAACATTGTCGTCGTCCCTAATGTGTACCGTACCTACTTGATATGTTTCCGCGGATTCCTAGGATCGAGCTCTATTTATTTATTTATTTTATATATATATATACATCTCTATATATATATG
```
eGFP | Pfpkg 3'UTR

6,380 6,400 6,420 6,440 6,460 6,480 6,500

```
TTAATTAGGGCGATATGATAATTGAAAGGATTATTATTTTTTAATTTTTTTAGTATATTTTTAGAAATCATATATATTCATAATAATATATTTGTTCCATCATATATATTATATATATATATATA
AATTAATCCCGCTATACTATTAACTTTCCTAATAATAAAAAATTAAAAAAATCATATAAAAATCTTTAGTATATATAAGTATTATTATATAAACAAGGTAGTATATATAATATATATATATATAT
```
Pfpkg 3'UTR

6,520 6,540 6,560 6,580 6,600 6,620

```
TTATATATATATATATAATAATTATAAATTTTTGAAAAATGTTATTTTTATTATTAATAATTTTTTTTTTTTTTTTTCATTTGTTTGTACAATATAATGAATTTATATCTTGCGGTGTTTTTTTTTAT
AATATATATATATATATTATTAATATTTAAAAACTTTTTACAATAAAAATAATAATTATTAAAAAAAAAAAAAAAAAGTAAACAAACATGTTATATTACTTAAATATAGAACGCCACAAAAAAAAATA
```
Pfpkg 3'UTR

6,640 6,660 6,680 6,700 6,720 6,740

```
TCTCTCCTTTTTTATATTATATTTTATTTTATTTTATTCTTTTTCTTTTTTTTTTTTTCTTTTTTTTTTTTTGTTTGTTTTTAATTTTACAAATATATTTAAATAAAAATTCAAATGGTTCTAAA
AGAGAGGAAAAAATATAATATAAAATAAAATAAAATAAGAAAAAGAAAAAAAAAAAAAGAAAAAAAAAAAAACAAACAAAAATTAAAATGTTTATATAAATTTATTTTTAAGTTTACCAAGATTT
```

Pfpkg 3'UTR

6,760 6,780 6,800 6,820 6,840 6,860

```
TTTTTTTAATAAATAAATAAAGTAATATTTACTATTTTAATTAGTGTTCAAAATGAAGAAGAATCAAAAAAAATGAAAATATGAAATTTGTATAAATATTTATAGGTATTTATATATATATATAT
AAAAAAATTATTTATTTATTTCATTATAAATGATAAAATTAATCACAAGTTTTACTTCTTCTTAGTTTTTTTTACTTTTATACTTTAAACATATTTATAAATATCCATAAATATATATATATATA
```

Pfpkg 3'UTR

6,880 6,900 6,920 6,940 6,960 6,980 7,000

```
ATATATATATATATATACACGGTGTTAATGCTTTACATTTTTTTTTTTTTTTTTCCTATGTCTATATATTTATTAATCCTTATATTTTTATATAAATTGTGCATT
TATATATATATATATATGTGCCACAATTACGAAATGTAAAAAAAAAAAAAAAAAGGATACAGATATATAAATAATTAGGAATATAAAAATATATTTAACACGTAA
```

Pfpkg 3'UTR

7,020 7,040 7,060 7,080 7,100

pDC_3cKO (6495 bp)

CcacTATTTTCTAACAGAATTAGTAACAGGTGGAGAATTATATGATGCTATTAGAAAATTAGGTTTATTATCTAAATCACAAGCTCAATTTTATTTAGGTTCTATCATTTTAGCTATTGAATATT
GgtgATAAAAGATTGTCTTAATCATTGTCCACCTCTTAATATACTACGATAATCTTTTAATCCAAATAATAGATTTAGTGTTCGAGTTAAAATAAATCCAAGATAGTAAAATCGATAACTTATAA

5' homology arm

20 40 60 80 100 120

TACATGAAAGAAATATTGTATATAGAGATTTAAAACCAGAAAACATTTTATTAGATAAACAAGGTTATGTAAAACTAATCGATTTTGGTTGTGCCAAAAAGGTACAAGGTAGAGCTTATACATTA
ATGTACTTTCTTTATAACATATATCTCTAAATTTTGGTCTTTTGTAAAATAATCTATTTGTTCCAATACATTTTGATTAGCTAAAACCAACACGGTTTTTCCATGTTCCATCTCGAATATGTAAT

5' homology arm

140 160 180 200 220 240

GTAGGTACACCTCATTATATGGCACCTGAGGTTATTTTAGGAAAAGGTTATGGATGTACTGTTGACATATGGGCATTGGGAATATGCCTATATGAATTTATATGTGGTCCATTACCATTTGGTAA
CATCCATGTGGAGTAATATACCGTGGACTCCAATAAAATCCTTTTCCAATACCTACATGACAACTGTATACCCGTAACCCTTATACGGATATACTTAAATATACACCAGGTAATGGTAAACCATT

5' homology arm

260 280 300 320 340 360

TGATGAAGAAGATCAATTAGAAATTTTCagaGATATTCTGACAGGCCAGCTGACCTTTCCGGATTATGTTACCGATACCGATAGCATCAACCTGATGAAACGCCTGCTGTGTCGTCTGCCGCAGG
ACTACTTCTTCTAGTTAATCTTTAAAAGtctCTATAAGACTGTCCGGTCGACTGGAAAGGCCTAATACAATGGCTATGGCTATCGTAGTTGGACTACTTTGCGGACGACACAGCAGACGGCGTCC

5' homology arm | PKGsynth fragment

380 400 420 440 460 480 500

GTCGTATTGGTTGTAGCATTAATGGTTTTAAAGACATCAAAGATCACCCGTTCTTCAGCAACTTTAACTGGGATAAACTGGCAGGTCGTCTGCTGGACCCTCCGCTGGTTAGCAAAAGCGAAACC
CAGCATAACCAACATCGTAATTACCAAAATTTCTGTAGTTTCTAGTGGGCAAGAAGTCGTTGAAATTGACCCTATTTGACCGTCCAGCAGACGACCTGGGAGGCGACCAATCGTTTTCGCTTTGG

PKGsynth fragment

520 540 560 580 600 620

TATGCAGAGGATATCGACATCAAACAAATCGAAGAAGAGGACGCAGAGGACGATGAGGAACCGCTGAATGACGAAGATAATTGGGATATCGATTTTTAAgaggttaccatcgagggatatggcag
ATACGTCTCCTATAGCTGTAGTTTGTTTAGCTTCTTCTCCTGCGTCTCCTGCTACTCCTTGGCGACTTACTGCTTCTATTAACCCTATAGCTAAAAATTctccaatggtagctccctataccgtc

PKGsynth fragment | PbDT 3'UTR

640 660 680 700 720 740

cttaatgttcgtttttcttatttatatatttataccaattgattgtatttataactgtaaaaatgtgtatgttgtgtgcatattttttttttgtgcatgcacatgcatgtaaatagctaaaattat
gaattacaagcaaaaagaataaatatataaatatggttaactaacataaatattgacatttttacacatacaacacacgtataaaaaaaaaacacgtacgtgtacgtacatttatcgattttaata

PbDT 3'UTR

760 780 800 820 840 860

gaacattttatttttttgttcagaaaaaaaaaactttacacacataaaatggctagtatgaatagccatattttatataaattaaatcctatgaatttatgaccatattaaaaatttagatattta
cttgtaaaataaaaaacaagtcttttttttttgaaatgtgtgtattttaccgatcatacttatcggtataaaatatatttaatttaggatacttaaatactggtataatttttaaatctataaat

PbDT 3'UTR

880 900 920 940 960 980 1,000

tggaacataatatgtttgaaacaataagacaaaattattattattattattattttttactgttataattatgtgtctccttcaatgattcataaatagttggacttgattttttaaaatgtttata
accttgtattatacaaactttgttattctgttttaataataataataataataaaaaatgacaatattaatacacagaggaagttactaagtatttatcaacctgaactaaaaattttacaaatat

PbDT 3'UTR

1,020 1,040 1,060 1,080 1,100 1,120

```
atatgattagcatagttaaataaaaaaagttgaaaaattaaaaaaaaacatataaacacaaatgatggtttttccttcaatttcgatatcatatagcaatcgtattagcaggtaaatgagcaagg
tatactaatcgtatcaatttatttttttcaactttttaatttttttttgtatatttgtgtttactaccaaaaaggaagttaaagctatagtatatcgttagcataatcgtccatttactcgttcc
                                                 PbDT 3'UTR
       1,140          1,160          1,180          1,200          1,220          1,240

ttaataaaaaaatggtaaaaaaaaaaaaattggggaaaaataaacaaggtgtctttatctacactttggctttacaatgtataatttatatgcatacaaaaaaatttttgataaaattgtaaaaagg
aattatttttttaccatttttttttttttaaccccttttttatttgttccacagaaatagatgtgaaaccgaaatgttacatattaaatatacgtatgttttttttaaaactattttaacatttttcc
                                                 PbDT 3'UTR
    1,260          1,280          1,300          1,320          1,340          1,360

tgaataaaataacattgtaacagtagtagatagtaaagggaaggtgttgctcaaatagtgtcgaaacaaaactggcataaagaaaaattgatattgagcagaggatatgcgcataatggtatttt
acttattttattgtaacattgtcatcatctatcatttcccttccacaacgagtttatcacagctttgttttgaccgtatttctttttaactataactcgtctcctatacgcgtattaccataaaa
                                                 PbDT 3'UTR
  1,380          1,400          1,420          1,440          1,460          1,480          1,500

gtttgtttgtttgttttctcatttttttgagacagctcaattctttatgtccacaacatcatcggacttttcttcttcagggtaggcGGCCGCTCGAGCATGCATCTAGATTTTATATAATAAAAA
caaacaaacaaacaaaagagtaaaaaaactctgtcgagttaagaaatacaggtgttgtagtagcctgaaaagaagaagtcccatccgCCGGCGAGCTCGTACGTAGATCTAAAATATATTATTTTT
                       PbDT 3'UTR
          1,520          1,540          1,560          1,580          1,600          1,620

ATGGAGAAGTAGAAATAGTAAAAAATAAAAAAAGGTTACgtgcggttAACAATAACTTCGTATAGCATACATTATACGAACGGTAtatatatgtatatatatatatatttatatattttatattc
TACCTCTTCATCTTTATCATTTTTTATTTTTTTCCAATGcacgccaaTTGTTATTGAAGCATATCGTATGTAATATGCTTGCCATatatatacatatatatatatataaatatataaaatataag
                                                  lox66                 SERA2 intron
       1,640          1,660          1,680          1,700          1,720          1,740

ttttagCGGCCGCCGTCTCCGGATCCGGTGGAGGCAGCGGAGGTGTTAGTAAAGGAGAAGAATTGATTAAAGAAAATATGCATATGAAATTGTATATGGAAGGAACAGTTGATAATCATCACTTC
aaaatcGCCGGCGGCAGAGGCCTAGGCCACCTCCGTCGCCTCCACAATCATTTCCTCTTCTTAACTAATTTCTTTTATACGTATACTTTAACATATACCTTCCTTGTCAACTATTAGTAGTGAAG
                                          BlueFP2 recodonised
    1,760          1,780          1,800          1,820          1,840          1,860

AAATGTACAAGTGAAGGAGAAGGAAAACCATATGAAGGAACACAAACAATGAGAATTAAAGTTGTTGAAGGAGGACCATTGCCATTTGCATTTGATATTTTGGCAACAAGTTTTTTGTATGGAAG
TTTACATGTTCACTTCCTCTTCCTTTTGGTATACTTCCTTGTGTTTGTTACTCTTAATTTCAACAACTTCCTCCTGGTAACGGTAAACGTAAACTATAAAACCGTTGTTCAAAAAACATACCTTC
                                          BlueFP2 recodonised
  1,880          1,900          1,920          1,940          1,960          1,980          2,000

TAAAACATTTATTAATCATACACAAGGAATTCCAGATTTTTTTAAACAAAGTTTTCCAGAAGGATTTACATGGGAAAGAGTTACAACATATGAAGATGGAGGAGTTTTGACAGCAACACAAGATA
ATTTTGTAAATAATTAGTATGTGTTCCTTAAGGTCTAAAAAAATTTGTTTCAAAAGGTCTTCCTAAATGTACCCTTTCTCAATGTTGTATACTTCTACCTCCTCAAAACTGTCGTTGTGTTCTAT
                                          BlueFP2 recodonised
          2,020          2,040          2,060          2,080          2,100          2,120

CAAGTTTGCAAGATGGATGTTTGATTTATAATGTTAAAATTAGAGGAGTTAATTTTACAAGTAATGGACCAGTTATGCAAAAAAAAACATTGGGATGGGAAGCATTTACAGAAACATTGTATCCA
GTTCAAACGTTCTACCTACAAACTAAATATTACAATTTTAATCTCCTCAATTAAAATGTTCATTACCTGGTCAATACGTTTTTTTTTGTAACCCTACCCTTCGTAAATGTCTTTGTAACATAGGT
                                          BlueFP2 recodonised
       2,140          2,160          2,180          2,200          2,220          2,240
```

```
GCAGATGGAGGATTGGAAGGAAGAAATGATATGGCATTGAAATTGGTTGGAGGAAGTCATTTGATTGCAAATGCAAAAACAACATATAGAAGTAAAAAACCAGCAAAAAATTTGAAAATGCCAGG
CGTCTACCTCCTAACCTTCCTTCTTTACTATACCGTAACTTTAACCAACCTCCTTCAGTAAACTAACGTTTACGTTTTTGTTGTATATCTTCATTTTTTGGTCGTTTTTTAAACTTTTACGGTCC
```

BlueFP2 recodonised

2,260 2,280 2,300 2,320 2,340 2,360

```
AGTTTATTATGTTGATTATAGATTGGAAAGAATTAAAGAAGCAAATAATGAAACATATGTTGAACAGCATGAAGTTGCAGTTGCAAGATATTGTGATTTGCCAAGTAAATTGGGACATAAATTGA
TCAAATAATACAACTAATATCTAACCTTTCTTAATTTCTTCGTTTATTACTTTGTATACAACTTGTCGTACTTCAACGTCAACGTTCTATAACACTAAACGGTTCATTTAACCCTGTATTTAACT
```

BlueFP2 recodonised

2,380 2,400 2,420 2,440 2,460 2,480 2,500

```
ATTAGGGCGATCCGCGGGGTACCCCATTAAATTTATTTAATAATAGATTAAAAATATTATAAAAATAAAAACATAAACACAGAAATTACAAAAAAAATACATATGAATTTTTTTTTTGTAATCTT
TAATCCCGCTAGGCGCCCCATGGGGTAATTTAAATAAATTATTATCTAATTTTTATAATATTTTTATTTTTGTATTTGTGTCTTTAATGTTTTTTTTATGTATACTTAAAAAAAAAACATTAGAA
```

hrp2 3'UTR

2,520 2,540 2,560 2,580 2,600 2,620

```
CCTTATAAATATAGAATAATGAATCATATAAAACATATCATTTTTCATTTATTTACATTTAAAATTATTGTTTCAGTATCTTTAATTTATTATGTATATATAAAAATAACTTACAATTTTATTAA
GGAATATTTATATCTTATTACTTAGTATATTTTGTATAGTAAAAAGTAAATAAATGTAAATTTTAATAACAAAGTCATAGAAATTAAATAATACATATATATTTTTATTGAATGTTAAAATAATT
```

hrp2 3'UTR

2,640 2,660 2,680 2,700 2,720 2,740

```
TAAACAATATATGTTTATTAATTCATGTTTTGTAATTTATGGGATAGCGATTTTTTTTACTGTCTGTATTTTTCTTTTTTAATTATGTTTTAATTGTATTTTATTTTTATTATTGTTCTTTTTAT
ATTTGTTATATACAAATAATTAAGTACAAAACATTAAATACCCTATCGCTAAAAAAAATGACAGACATAAAAAGAAAAAATTAATACAAAATTAACATAAAATAAAAATAATAACAAGAAAAATA
```

hrp2 3'UTR

2,760 2,780 2,800 2,820 2,840 2,860

```
AGTATTATTTTAAAACAAAATGTTTTTTCTAAGAACTTATAATAATAATAAATATAAATTTTAATAAAAAATTATATTTATCTTTTACAATATGAACATAAAGTACAACATTAATATATAGCTTTT
TCATAATAAAATTTTGTTTTACAAAAAAGATTCTTGAATATTATTATTATTTATATTTAAAATTATTTTTAATATAAATAGAAAATGTTATACTTGTATTTCATGTTGTAATTATATATCGAAAA
```

hrp2 3'UTR

2,880 2,900 2,920 2,940 2,960 2,980 3,000

```
AATATTTTTATTCCTAATCATGTAAATCTTAAATTTTTCTTTTTAAACATATGTTAAATATTTATTTCTCATTATATATAAGGACATATTTATTAAACCGCAGAGAAATCTAGAGGTACCGAGCT
TTATAAAAATAAGGATTAGTACATTTAGAATTTAAAAAGAAAAATTTGTATACAATTTATAAATAAAGAGTAATATATATTCCTGTATAAATAATTTGGCGTCTCTTTAGATCTCCATGGCTCGA
```

hrp2 3'UTR

3,020 3,040 3,060 3,080 3,100 3,120

```
AATGGAGAAGTAGAAATAGTACATAACTTCGTATAGTATACCTTATACGAAGTTATtatatatgtatatatatatatatttatatatttatattcttttagAGATGTCTGTGAGTAAAGGAGAA
TTACCTCTTCATCTTTATCATGTATTGAAGCATATCATATGGAATATGCTTCAATAatatatacatatatatatatataaatatataaaatataagaaaatcTCTACAGACACTCATTTCCTCTT
```

loxN SERA2 intron eGFP

3,140 3,160 3,180 3,200 3,220 3,240

```
GAACTTTTCACTGGAGTTGTCCCAATTCTTGTTGAATTAGATGGTGATGTTAATGGGCACAAATTTTCTGTCAGTGGAGAGGGTGAAGGTGATGCAACATACGGAAAACTTACCCTTAAATTTAT
CTTGAAAAGTGACCTCAACAGGGTTAAGAACAACTTAATCTACCACTACAATTACCCGTGTTTAAAAGACAGTCACCTCTCCCACTTCCACTACGTTGTATGCCTTTTGAATGGGAATTTAAATA
```

eGFP

3,260 3,280 3,300 3,320 3,340 3,360

TTGCACTACTGGAAAACTACCTGTTCCCTGGCCAACACTTGTCACTACTTTCGGTTATGGTGTTCAATGCTTTGCGAGATACCCAGATCACATGAAACAGCATGACTTTTTCAAGAGTGCCATGC
AACGTGATGACCTTTTGATGGACAAGGGACCGGTTGTGAACAGTGATGAAAGCCAATACCACAAGTTACGAAACGCTCTATGGGTCTAGTGTACTTTGTCGTACTGAAAAAGTTCTCACGGTACG

eGFP

3,380 3,400 3,420 3,440 3,460 3,480 3,500

CCGAAGGTTATGTACAGGAAAGAACTATATTTTTCAAAGATGACGGGAACTACAAGACACGCGCTGAAGTCAAGTTTGAAGGTGATACCCTTGTTAATAGAATCGAGTTAAAAGGTATTGATTTT
GGCTTCCAATACATGTCCTTTCTTGATATAAAAAGTTTCTACTGCCCTTGATGTTCTGTGCGCGACTTCAGTTCAAACTTCCACTATGGGAACAATTATCTTAGCTCAATTTTCCATAACTAAAA

eGFP

3,520 3,540 3,560 3,580 3,600 3,620

AAAGAAGATGGAAACATTCTTGGACACAAATTGGAATACAACTATAACTCACACAATGTTTACATCATGGCAGACAAACAAAAGAATGGAATCAAAGTAAACTTCAAAATTAGACACAACATTGA
TTTCTTCTACCTTTGTAAGAACCTGTGTTTAACCTTATGTTGATATTGAGTGTGTTACAAATGTAGTACCGTCTGTTTGTTTTCTTACCTTAGTTTCATTTGAAGTTTTAATCTGTGTTGTAACT

eGFP

3,640 3,660 3,680 3,700 3,720 3,740

AGATGGAAGCGTTCAACTAGCAGACCATTATCAACAAAATACTCCAATTGGCGATGGCCCTGTCCTTTTACCAGACAACCATTACCTGTCCACACAATCTGCCCTATCGAAAGATCCCAACGAAA
TCTACCTTCGCAAGTTGATCGTCTGGTAATAGTTGTTTTATGAGGTTAACCGCTACCGGGACAGGAAAATGGTCTGTTGGTAATGGACAGGTGTGTTAGACGGGATAGCTTTCTAGGGTTGCTTT

eGFP

3,760 3,780 3,800 3,820 3,840 3,860

AGAGAGACCACATGGTCCTTCTTGAGTTTGTAACAGCAGCAGGGATTACACATGGCATGGATGAACTATACAAAGGCGCCTAATCGAGATCCTAGCTCGAGTTATATAATATATTTATGTACTCA
TCTCTCTGGTGTACCAGGAAGAACTCAAACATTGTCGTCGTCCCTAATGTGTACCGTACCTACTTGATATGTTTCCGCGGATTAGCTCTAGGATCGAGCTCAATATATTATATAAATACATGAGT

eGFP hsp86 3'

3,880 3,900 3,920 3,940 3,960 3,980 4,000

CAATGGGGTCTACAAAAAAAAAAAAATAAAGATATATATATATATATATTATCTTTCATTTTATCATCACCCCAATATACATATATATATTTTTATATTTTTATATATTTATATTTTTCTTTTAT
GTTACCCCAGATGTTTTTTTTTTTTTATTTCTATATATATATATATATAATAGAAAGTAAAATAGTAGTGGGGTTATATGTATATATATATAAAAATATAAAAATATATAAATATAAAAAGAAAATA

hsp86 3'

4,020 4,040 4,060 4,080 4,100 4,120

TTATCCATAAAAGAAAATTAAATATAAATTTATACCTTTTATTTGAATTACCATTTGTATAAAATATAAAAAAAAAAAAAAATAATAAAAAGAAGAAAAAACGAAAAAAGTAATATTTAAATAAT
AATAGGTATTTTCTTTTAATTTATATTTAAATATGGAAAATAAACTTAATGGTAAACATATTTTATATTTTTTTTTTTTTTTATTATTTTTCTTCTTTTTTGCTTTTTTCATTATAAATTTATTA

hsp86 3'

4,140 4,160 4,180 4,200 4,220 4,240

AAAGATCAATATACATAATAAAAAAAAAAAAAAAAAAAAAATTAAAATTTAAAAAAATTAAACATTGAAATAATTTCATATACACACATATACATACATACATATATATATATATATATATATAT
TTTCTAGTTATATGTATTATTTTTTTTTTTTTTTTTTTTTTAATTTTAAATTTTTTTAATTTGTAACTTTATTAAAGTATATGTGTGTATATGTATGTATGTATATATATATATATATATATATA

hsp86 3'

4,260 4,280 4,300 4,320 4,340 4,360

TTATACATTTATGCATATAAAATATATTAACAACTTATGTGTATTCATATATATTTTTTTACATATGTATTGAAAGACTTTACTGAGACATGAAATATCTAATAACCAAACGTTTATAAATTCTA
AATATGTAAATACGTATATTTTATATAATTGTTGAATACACATAAGTATATATAAAAAAATGTATACATAACTTTCTGAAATGACTCTGTACTTTATAGATTATTGGTTTGCAAATATTTAAGAT

hsp86 3'

4,380 4,400 4,420 4,440 4,460 4,480 4,500

```
ATTATATGTAAAGCATAATATAGCTTTTGTCTAATTTAATTCCTTAAAACGTATGTATTAAAAATATAGTAAGATGGGGAAATTATAATTTTTTTTTTAAAGGTTTTTCTTTGATACAGTAAAAA
TAATATACATTTCGTATTATATCGAAAACAGATTAAATTAAGGAATTTTGCATACATAATTTTTATATCATTCTACCCCTTTAATATTAAAAAAAAAATTTCCAAAAAGAAACTATGTCATTTTT
hsp86 3'
      4,520     4,540     4,560     4,580     4,600     4,620

AAAAAAATAAAAAAAAATAATAATAATAAAAAAATAAAAAAATAAAAAAAAATAAAATAATTATAAAGATATATATAATTCCTCCCAAATACAAATGATTAAGTTTAAAAATTAATAAATAAATC
TTTTTTTATTTTTTTTTATTATTATTATTTTTTTATTTTTTTATTTTTTTTTATTTTATTAATATTTCTATATATATTAAGGAGGGTTTATGTTTACTAATTCAAATTTTTAATTATTTATTTAG
hsp86 3'
      4,640     4,660     4,680     4,700     4,720     4,740

AAAAAAAAATTTAATAATAATTGTATTATTTTAAGTGTAGTTAATTCATCAAATAGCATGCCTGCAGGTCGACagtcTCCGGActcgGCTAGCcgatTACAACCATTTTGTAAAAAAAATTAAAA
TTTTTTTTTAAATTATTATTAACATAATAAAATTCACATCAATTAAGTAGTTTATCGTACGGACGTCCAGCTGtcagAGGCCTgagcCGATCGgctaATGTTGGTAAAACATTTTTTTTAATTTT
hsp86 3'
      4,760     4,780     4,800     4,820     4,840     4,860

TATATTTATATAATATTATTTTATTTTATTATATATTATATTATTTTTATTTTTATTTTTATTTTTTTTTCTCTACAAATTTTATCTATTGGTTTATTATAAAAATATCTATTTCTAATAATAAA
ATATAAATATATTATAATAAAATAAAATAATATATAATATAATAAAAATAAAAATAAAAATAAAAAAAAAGAGATGTTTAAAATAGATAACCAAATAATATTTTTATAGATAAAGATTATTATTT
      4,880     4,900     4,920     4,940     4,960     4,980     5,000

TAATTAAGATATCAAGCGGCCTTCCGCGAATAACTTCGTATAGGATACTTTATACGAAGTTATTATATATGTATATATATATATATTTATATATTTTATATTCTTTTAGGGTCACCTAGGATGAA
ATTAATTCTATAGTTCGCCGGAAGGCGCTTATTGAAGCATATCCTATGAAATATGCTTCAATAATATATACATATATATATATATAAATATATAAAATATAAGAAAATCCCAGTGGATCCTACTT
lox2272   SERA2 intron
      5,020     5,040     5,060     5,080     5,100     5,120

GGTGAGCAAGGGCGAGGAGGATAACATGGccatcatcaaggagttcatgcgcttcaaggtgcacatggagggctccgtgaacggccacgagttcgagatcgagggcgagggcgagggccgcccct
CCACTCGTTCCCGCTCCTCCTATTGTACCggtagtagttcctcaagtacgcgaagttccacgtgtacctcccgaggcacttgccggtgctcaagctctagctcccgctcccgctcccggcgggga
mCherry
      5,140     5,160     5,180     5,200     5,220     5,240

acgagggcacccagaccgccaagctgaaggtgaccaagggtggccccctgcccttcgcctgggacatcctgtcccctcagttcatgtacggctccaaggcctacgtgaagcaccccgccgacatc
tgctcccgtgggtctggcggttcgacttccactggttcccaccgggggacgggaagcggaccctgtaggacaggggagtcaagtacatgccgaggttccggatgcacttcgtggggcggctgtag
mCherry
      5,260     5,280     5,300     5,320     5,340     5,360

cccgactacttgaagctgtccttccccgagggcttcaagtgggagcgcgtgatgaacttcgaggacggcggcgtggtgaccgtgacccaggactcctccctgcaggacggcgagttcatctacaa
gggctgatgaacttcgacaggaaggggctcccgaagttcaccctcgcgcactacttgaagctcctgccgccgcaccactggcactgggtcctgaggagggacgtcctgccgctcaagtagatgtt
mCherry
      5,380     5,400     5,420     5,440     5,460     5,480     5,500

ggtgaagctgcgcggcaccaacttcccctccgacggccccgtaatgcagaagaagaccatgggctgggaggcctcctccgagcggatgtaccccgaggacggcgccctgaagggcgagatcaagc
ccacttcgacgcgccgtggttgaaggggaggctgccggggcattacgtcttcttctggtacccgaccctccggaggaggctcgcctacatggggctcctgccgcgggacttcccgctctagttcg
mCherry
      5,520     5,540     5,560     5,580     5,600     5,620
```

agaggctgaagctgaaggacggcggccactacgacgctgaggtcaagaccacctacaaggccaagaagcccgtgcagctgcccggcgcctacaacgtcaacatcaagttggacatcacctcccac
tctccgacttcgacttcctgccgccggtgatgctgcgactccagttctggtggatgttccggttcttcgggcacgtcgacgggccgcggatgttgcagttgtagttcaacctgtagtggagggtg

mCherry

5,640 5,660 5,680 5,700 5,720 5,740

aacgaggactacaccatcgtggaacagtacgaacgcgccgagggccgccactccaccggcggcatggacgagctgtacaagtaacTCGAGATAAATAAATAAATAAAATATATATATATATGTAG
ttgctcctgatgtggtagcaccttgtcatgcttgcgcggctcccggcggtgaggtggccgccgtacctgctcgacatgttcattgAGCTCTATTTATTTATTTATTTTATATATATATATATACATC

mCherry Pfpkg 3’UTR

5,760 5,780 5,800 5,820 5,840 5,860

AGATATATATATACTTAATTAGGGCGATATGATAATTGAAAGGATTATTATTTTTTAATTTTTTTAGTATATTTTTAGAAATCATATATATTCATAATAATATATTTGTTCCATCATATATATTA
TCTATATATATATGAATTAATCCCGCTATACTATTAACTTTCCTAATAATAAAAAATTAAAAAAATCATATAAAAATCTTTAGTATATATAAGTATTATTATATAAACAAGGTAGTATATATAAT

Pfpkg 3’UTR

5,880 5,900 5,920 5,940 5,960 5,980 6,000

TATATATATATATATTATATATATATATAATAATTATAAATTTTTGAAAAATGTTATTTTTATTATTAATAATTTTTTTTTTTTTTTTCATTTGTTTGTACAATATAATGAATTTATATCTTGCG
ATATATATATATATATAATATATATATATATTATTAATATTTAAAAACTTTTTACAATAAAAATAATAATTATTAAAAAAAAAAAAAAAAGTAAACAAACATGTTATATTACTTAAATATAGAACGC

Pfpkg 3’UTR

6,020 6,040 6,060 6,080 6,100 6,120

GTGTTTTTTTTTATTCTCTCCTTTTTTATATTATATTTTATTTTATTTTATTCTTTTTCTTTTTTTTTTTTTCTTTTTTTTTTTTTGTTTGTTTTTAATTTTACAAATATATTTAAATAAAAATT
CACAAAAAAAAATAAGAGAGGAAAAAATATAATATAAAATAAAATAAAATAAGAAAAAGAAAAAAAAAAAAAGAAAAAAAAAAAAACAAACAAAAATTAAAATGTTTATATAAATTTATTTTTAA

Pfpkg 3’UTR

6,140 6,160 6,180 6,200 6,220 6,240

CAAATGGTTCTAAATTTTTTTAATAAATAAATAAAGTAATATTTACTATTTTAATTAGTGTTCAAAATGAAGAAGAATCAAAAAAAAATGAAAATATGAAATTTGTATAAATATTTATAGGTATTT
GTTTACCAAGATTTAAAAAAATTATTTATTTATTTCATTATAAATGATAAAATTAATCACAAGTTTTACTTCTTCTTAGTTTTTTTTTACTTTTATACTTTAAACATATTTATAAATATCCATAAA

Pfpkg 3’UTR

6,260 6,280 6,300 6,320 6,340 6,360

ATATATATATATATATATATATATATATATATACACGGTGTTAATGCTTTACATTTTTTTTTTTTTTTTTTCCTATGTCTATATATTTATTAATCCTTATATTTTTATATAAATTGTGCATTA
TATATATATATATATATATATATATATATATATGTGCCACAATTACGAAATGTAAAAAAAAAAAAAAAAAAGGATACAGATATATAAATAATTAGGAATATAAAAATATATTTAACACGTAAT

Pfpkg 3’UTR

6,380 6,400 6,420 6,440 6,460 6,480

pMX_3lox (1057 bp)

```
gGAATGAAAGAAATAAAAAGAAGGCTATATTTTCAAATGATGATTTTACAGGAGAAGATAGTTTAATGGAGgtaagaaataaaaggaatttgtaattttttttttaaattgtatatttaaaaggg
cCTTACTTTCTTTATTTTTCTTCCGATATAAAAGTTTACTACTAAAATGTCCTCTTCTATCAAATTACCTCcattctttattttccttaaacattaaaaaaaaaatttaacatataaattttccc
```

5' homology arm

20 40 60 80 100 120

```
aataatgtatgtattaattttgtgatcgtttattatatattatttttaaattacatatccatatttaattatatacaaatgttgttttcagaattttttttttttttttttttctatttatctat
ttattacatacataattaaaacactagcaaataatatataataaaaatttaatgtataggtataaattaatatatgtttacaacaaaagtcttaaaaaaaaaaaaaaaaaaaagataaatagata
```

5' homology arm

140 160 180 200 220 240

```
aacatatatttaattttatttttagGATCATTTAGAACTTCGGGAAAAGCTTTCAGAAGATATTGATATGATAAAGACTTCCTTAAAAAATAATCTAGTTTGTAGTACATTAAACGATAATGAAA
ttgtatataaattaaaataaaaatcCTAGTAAATCTTGAAGCCCTTTTCGAAAGTCTTCTATAACTATACTATTTCTGAAGGAATTTTTTATTAGATCAAACATCATGTAATTTGCTATTACTTT
```

5' homology arm

260 280 300 320 340 360

```
TATTGACTCTGTCTAATTATATGCAATTCTTTGTTTTTAAAAGTGGAAATTTAGTAATAAAACAAGGGGAAAAAGgtaaataaaaaaaaTACCGTTCGTATAGCATACATTATACGAAGTTATta
ATAACTGAGACAGATTAATATACGTTAAGAAACAAAAATTTTCACCTTTAAATCATTATTTTGTTCCCCTTTTTCcatttattttttttATGGCAAGCATATCGTATGTAATATGCTTCAATAat
```

lox71

5' homology arm

380 400 420 440 460 480 500

```
atatacaATAACTTCGTATAGTATACCTTATACGAAGTTATTATATATGTATATATATAATAACTTCGTATAGGATACTTTATACGAAGTTATtatatttatatatttatattcttttagGGTC
tatatgtTATTGAAGCATATCATATGGAATATGCTTCAATAATATATACATATATATATTATTGAAGCATATCCTATGAAATATGCTTCAATAatataaatatataaatataagaaaatcCCAG
```

loxN lox2272

3loxPint (sera2 intron with lox71, loxN and lox2272)

520 540 560 580 600 620

```
ATACTTTTTCATTATTAATAGTGGCAAATTTGACGTTTATGTAAATGATAAAAAAGTAAAGACTATGGGAAAAGGTAGTTCTTTCGGTGAAGCTGCTTTAATTCATAATACCCAAAGAAGTGCAA
TATGAAAAAGTAATAATTATCACCGTTTAAACTGCAAATACATTTACTATTTTTTCATTTCTGATACCCTTTTCCATCAAGAAAGCCACTTCGACGAAATTAAGTATTATGGGTTTCTTCACGTT
```

3' homology arm

640 660 680 700 720 740

```
CTATTATTGCAGAAACTGATGGAACTCTATGGGGAGTTCAAAGAAGTACATTTAGAGCTACCCTAAAACAATTATCTAATAGAAATTTTAACGAAAACAGAACATTTATCGATTCCGTTTCAGTT
GATAATAACGTCTTTGACTACCTTGAGATACCCCTCAAGTTTCTTCATGTAAATCTCGATGGGATTTTGTTAATAGATTATCTTTAAAATTGCTTTTGTCTTGTAAATAGCTAAGGCAAAGTCAA
```

3' homology arm

760 780 800 820 840 860

```
TTTGATATGTTAACTGAAGCACAAAAAAACATGATTACTAATGCTTGTGTAATACAAAACTTTAAATCTGGTGAAACCATTGTTAAACAAGGAGATTATGGAGATGTCTTATACATTTTGAAAGA
AAACTATACAATTGACTTCGTGTTTTTTTGTACTAATGATTACGAACACATTATGTTTTGAAATTTAGACCACTTTGGTAACAATTTGTTCCTCTAATACCTCTACAGAATATGTAAAACTTTCT
```

3' homology arm

880 900 920 940 960 980 1,000

```
AGGAAAGGCTACAGTATATATTAACGATGAAGAGATAAGGGTTTTAGAGAAAgtacg
TCCTTTCCGATGTCATATATAATTGCTACTTCTCTATTCCCAAAATCTCTTTcatgc
```

3' homology arm

1,020 1,040
