# Supplementary material for: Simultaneous multiple allelic replacement in the malaria parasite enables dissection of PKG function
Source: Life Sci Alliance. 2020 Mar 16;3(4):e201900626. doi: 10.26508/lsa.201900626 (PMC7081069; doi:10.26508/lsa.201900626)
Supplement: Supplementary file 4 [file LSA-2019-00626_TableS1.docx]

**Table S1: Oligonucleotide primers used in this study:**

| **Name** | **Sequence** |
| --- | --- |
| exon1_For | GAAGAAGATGATAATCTAAAAAAAGGG |
| Intron3_Rev | GTATGACCCTAAAAGGGGGG |
| exon4_Rev | gctctaaatgtacttctttgaactcc |
| wtpkg_For | CTGGTGAAACCATTGTTAAACAAGG |
| 5int_Rev | CTATTTACATGCATGTGCATGCAC |
| PKGsynth_For | CCGGTGGTGAACTGTATGACGC |
| 3int_Rev | GGTCATGTATGTTTAGAACCTGTAC |
| PKGutr_Rev | CCTTTCAATTATCATATCGCCC |
| bfp_Rev | CCCAATTTACTTGGCAAATCAC |
| gfp_Rev | CGTATGTTGCATCACCTTCAC |
| mCherry_Rev | GAACTCCTTGATGATGGCCATG |
